# Excess Patient Visits for Cough and Pulmonary Disease at a Large US Health System in the Months Prior to the COVID-19 Pandemic: Time-Series Analysis

**DOI:** 10.2196/21562

**Published:** 2020-09-10

**Authors:** Joann G Elmore, Pin-Chieh Wang, Kathleen F Kerr, David L Schriger, Douglas E Morrison, Ron Brookmeyer, Michael A Pfeffer, Thomas H Payne, Judith S Currier

**Affiliations:** 1 Department of Medicine David Geffen School of Medicine UCLA Los Angeles, CA United States; 2 Department of Biostatistics UW School of Public Health Seattle, WA United States; 3 Department of Emergency Medicine David Geffen School of Medicine UCLA Los Angeles, CA United States; 4 Department of Biostatistics Fielding School of Public Health UCLA Los Angeles, CA United States; 5 Department of Medicine UW School of Medicine Seattle, WA United States

**Keywords:** COVID-19, pandemic, electronic health record, time-series analysis, prediction, forecast

## Abstract

**Background:**

Accurately assessing the regional activity of diseases such as COVID-19 is important in guiding public health interventions. Leveraging electronic health records (EHRs) to monitor outpatient clinical encounters may lead to the identification of emerging outbreaks.

**Objective:**

The aim of this study is to investigate whether excess visits where the word “cough” was present in the EHR reason for visit, and hospitalizations with acute respiratory failure were more frequent from December 2019 to February 2020 compared with the preceding 5 years.

**Methods:**

A retrospective observational cohort was identified from a large US health system with 3 hospitals, over 180 clinics, and 2.5 million patient encounters annually. Data from patient encounters from July 1, 2014, to February 29, 2020, were included. Seasonal autoregressive integrated moving average (SARIMA) time-series models were used to evaluate if the observed winter 2019/2020 rates were higher than the forecast 95% prediction intervals. The estimated excess number of visits and hospitalizations in winter 2019/2020 were calculated compared to previous seasons.

**Results:**

The percentage of patients presenting with an EHR reason for visit containing the word “cough” to clinics exceeded the 95% prediction interval the week of December 22, 2019, and was consistently above the 95% prediction interval all 10 weeks through the end of February 2020. Similar trends were noted for emergency department visits and hospitalizations starting December 22, 2019, where observed data exceeded the 95% prediction interval in 6 and 7 of the 10 weeks, respectively. The estimated excess over the 3-month 2019/2020 winter season, obtained by either subtracting the maximum or subtracting the average of the five previous seasons from the current season, was 1.6 or 2.0 excess visits for cough per 1000 outpatient visits, 11.0 or 19.2 excess visits for cough per 1000 emergency department visits, and 21.4 or 39.1 excess visits per 1000 hospitalizations with acute respiratory failure, respectively. The total numbers of excess cases above the 95% predicted forecast interval were 168 cases in the outpatient clinics, 56 cases for the emergency department, and 18 hospitalized with acute respiratory failure.

**Conclusions:**

A significantly higher number of patients with respiratory complaints and diseases starting in late December 2019 and continuing through February 2020 suggests community spread of SARS-CoV-2 prior to established clinical awareness and testing capabilities. This provides a case example of how health system analytics combined with EHR data can provide powerful and agile tools for identifying when future trends in patient populations are outside of the expected ranges.

## Introduction

Health systems, medical providers, bioinformaticians, and researchers worldwide are working tirelessly to understand, contain, and ameliorate the COVID-19 pandemic. During this health emergency, clinicians have anecdotally noted an unusual number of patients with respiratory complaints at the end of 2019 and early 2020, well before COVID-19 was officially categorized by the World Health Organization (WHO) as a pandemic [1]. It is unclear whether such anecdotal reports are correct or the result of hindsight bias. If correct, the excess could represent typical variation in disease patterns. Alternatively, the excess, especially if it is significantly above prediction intervals based on historical data, could represent undetected and early COVID-19 cases prior to established clinical awareness and testing capabilities for the virus.

In the past decade, there has been widespread adoption of electronic health records (EHRs) in the United States. However, there have been limited efforts to date to leverage EHRs to support the delivery of high-value medical care or otherwise improve the delivery of health care services [2]. Using EHR data to model and forecast trends has the potential to improve resource management and the preparedness of health systems [3-7], which in turn could improve the quality of medical care. In particular, EHR data paired with analytical tools can potentially identify unusual trends in health care delivery that can alert clinicians and public health experts to critical changes in disease patterns.

The purpose of this paper is to use discrete raw EHR data to evaluate whether there was an excess of patients presenting with symptoms and diseases suggestive of COVID-19 in the months prior to the first known COVID-19 cases in the US health system in March 2020, using words found in chief complaint fields and International Classification of Diseases (ICD) codes from hospital discharge diagnoses. We analyzed 5 years of data from a large Los Angeles–area health system using time-series methods to address whether there was an excess number of patients presenting for complaints of cough, or hospitalizations for respiratory ailments. These methods highlight how health care analytics coupled with EHR data can be harnessed for disease surveillance. In particular, surveillance starting from the larger outpatient setting, which is often the tip of the iceberg, can provide an early warning of a public health emergency before patients fill hospital intensive care units and deaths accumulate.

## Methods

The study included data from July 1, 2014, to February 29, 2020, from UCLA Health, a large health system with over 2.5 million total outpatient visits and 3 hospitals (UCLA Medical Center Santa Monica, Resnick Neuropsychiatric Hospital at UCLA, and Ronald Reagan UCLA Medical Center). Health system utilization data during the winter season, from December 1, 2019, to February 29, 2020, the months prior to increased public awareness of COVID-19 in the United States, were evaluated using the previous 5 years as the comparison period in a time-series analysis. Data were collected using SQL reports from Epic Clarity production databases supporting the EHRs used throughout UCLA Health.

Analyses included three different care settings: outpatient clinics, emergency departments, and hospital. All primary, specialty, and urgent care outpatient visits were considered and searched for the word “cough” within the reason for visit, further examining the percentages of patients presenting with cough in the current winter season with forecast predictions based on data for the preceding 5 years. All patient visits to emergency departments for cough and data on patients hospitalized with acute respiratory failure for the current winter season were separately examined with the corresponding data for recent years in the same manner (see Table 7 in Multimedia Appendix 1 for a list of ICD codes).

**Figure 1:**
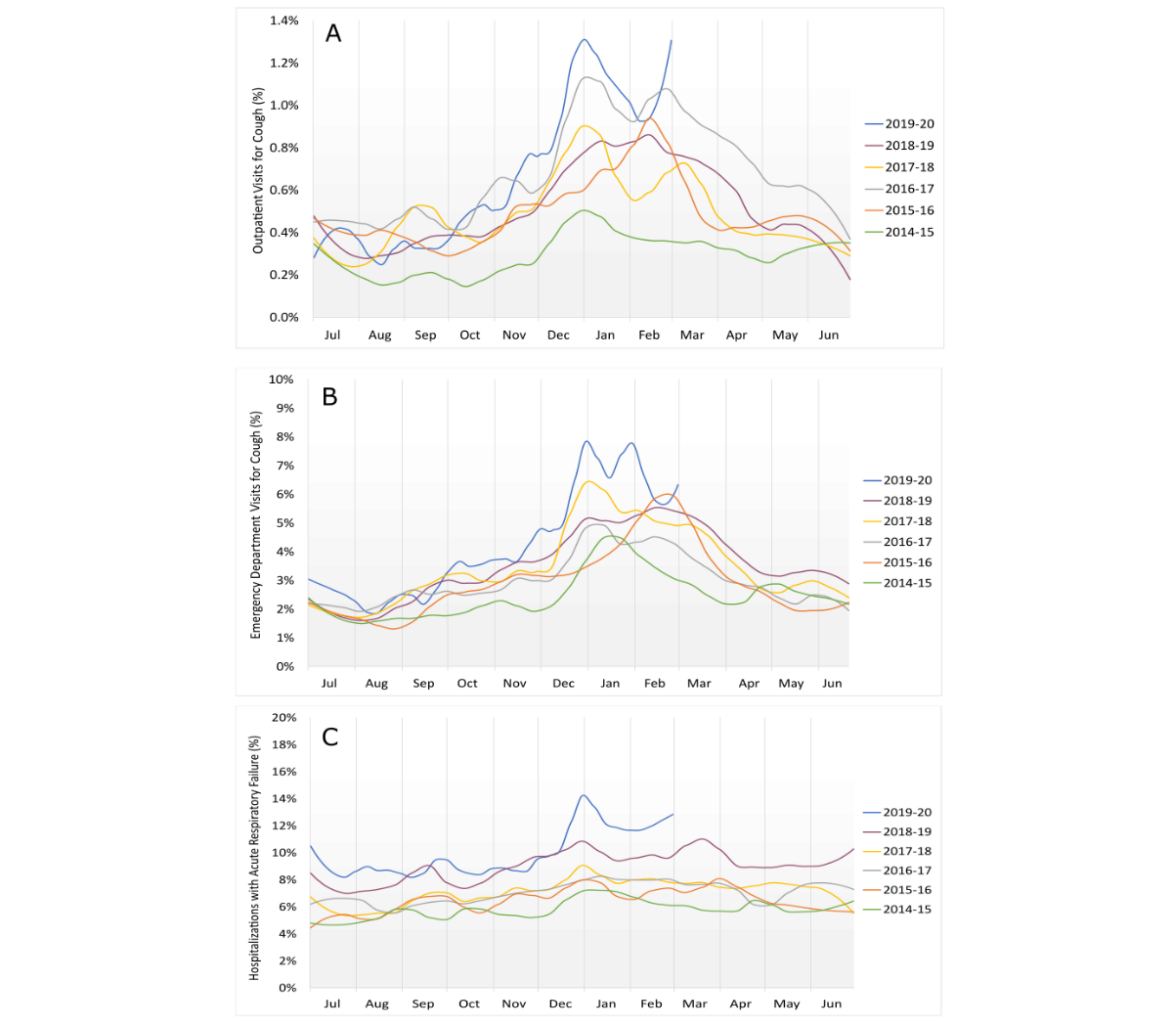
Percentage of outpatient and emergency department visits for cough and hospitalizations for acute respiratory failure from July 1, 2014, to February 29, 2020. Vertical reference lines align to the first day of each month. (A) Outpatient clinic visits for complaints of cough. (B) Emergency department visits for cough. (C) Hospitalizations for acute respiratory failure.

**Figure 2:**
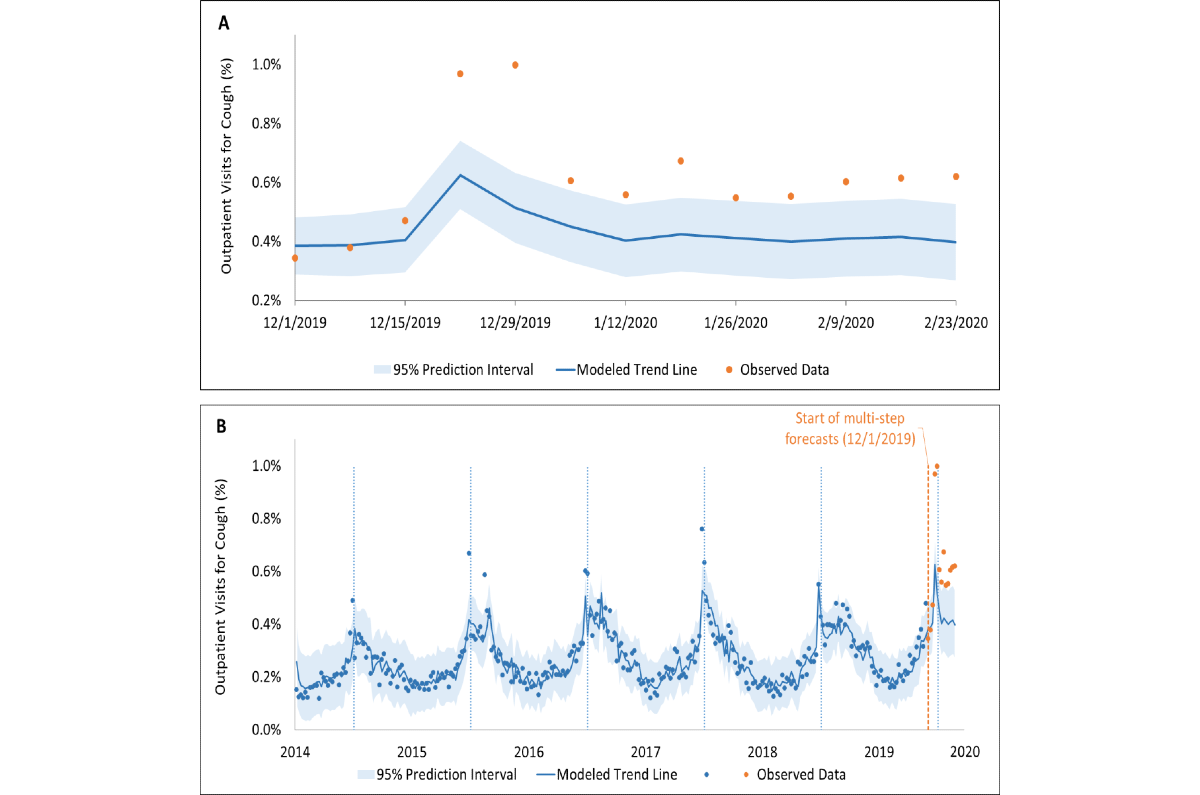
Time series analysis of outpatient data. (A) Forecast, with 95% prediction intervals, of expected rates of outpatient presentations for cough (based on time-series analyses of the previous 5 years), with observed data for each week shown for December 1, 2019, to February 29, 2020. (B) Time-series analysis of outpatient data.

**Figure 3:**
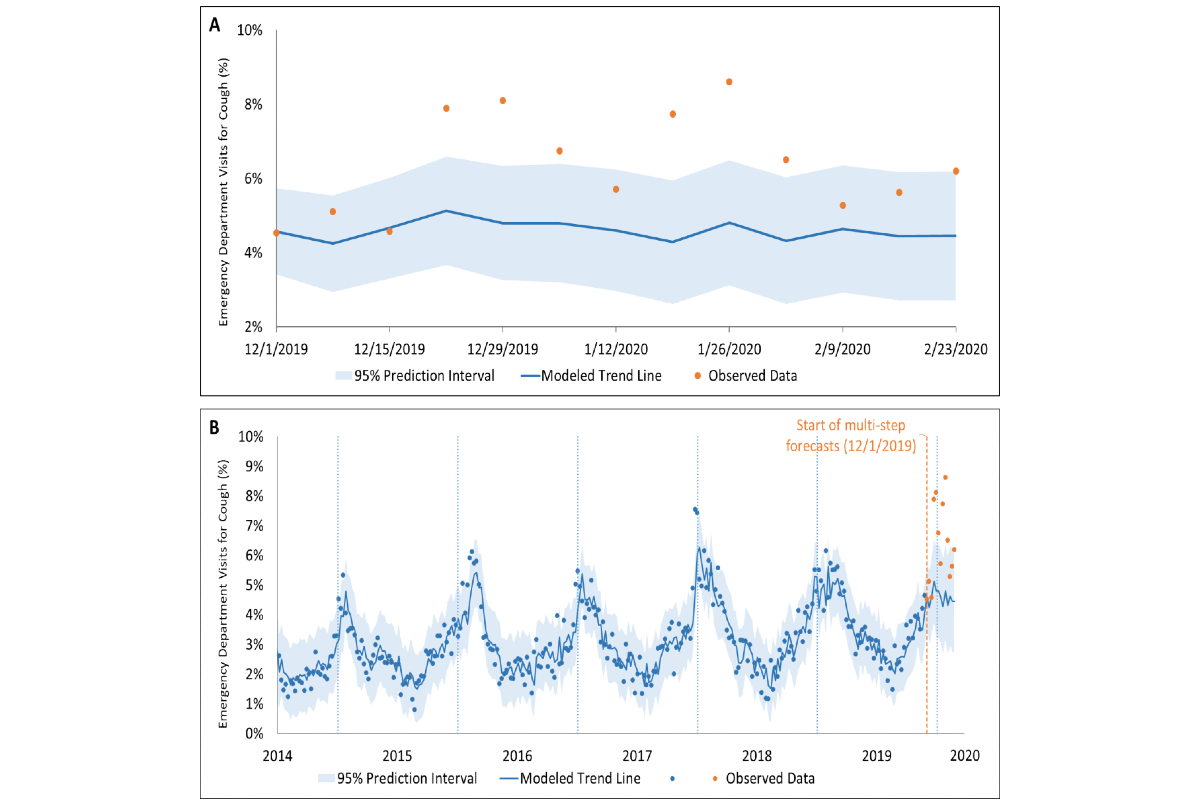
Time-series analysis of emergency department visits for cough. (A) Forecast, with 95% prediction intervals, of expected rates of emergency department presentations for cough (based on time-series analyses of the previous 5 years), with observed data for each week shown for December 1, 2019, to February 29, 2020. (B) Time-series analysis of emergency department data.

Seasonal autoregressive integrated moving average (SARIMA) models were applied on weekly data in SAS (SAS Institute) from July 1, 2014, through November 30, 2019, to forecast data for December 2019 through February 2020 (Tables 1-6 and Figures 1-3 in Multimedia Appendix 1). These models take into account seasonal effects [8,9] and use maximum likelihood to estimate model parameters based on historical data from July 1, 2014, to November 30, 2019. A winter season is defined as the time period from December 1 to the last day of February the subsequent year. Using the SARIMA model, a forecast of the 2019/2020 winter season was provided. Specifically, a SARIMA(1,0,1)x(1,0,1)_52_ model was used to analyze the outpatient time series, a SARIMA(1,0,3)x(1,0,1)_52_ model was used to analyze the emergency department data, and a SARIMA(1,0,1)x(1,0,1)_52_ model was used to analyze the inpatient data for patients with acute respiratory failure. The autoregressive and moving-average orders were identified by both SCAN and extended sample autocorrelation function (ESACF) methods [10].

The 95% prediction intervals for the forecast allowed an assessment of whether the observed data at weekly intervals for the 2019/2020 winter season were outside of the time-series prediction interval.

For example, for the outpatient clinic visit data, the following SARIMA(1,0,1)x(1,0,1)_52_ model was used (model parameter estimates are shown in Table 1 in Multimedia Appendix 1):



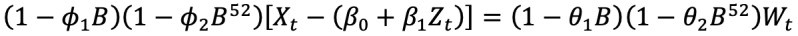



Where *X_t_* is the percentage of visits in week for which cough was a recorded symptom; *B* is the backshift operator, *BX_t_* = *X_t_*_-1_; 
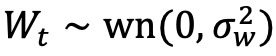
 is a white noise process of uncorrelated random variables with mean 0 and variance 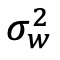; and *Z_t_* is an indicator variable for the months of December to February.

Excess cases per 1000 visits for the 2019/2020 winter season compared to previous seasons were estimated using three methods (Tables 8-11 in Multimedia Appendix 1). First, for a conservative estimate of excess cases, the maximum of the five previous seasons was subtracted from the current season. Second, the average of the five previous seasons was subtracted from the current season. For both methods, the excess percentage was multiplied by the total number of patient visits in the current season to estimate the excess cases for each month. For the third method, the upper limit of the time-series 95% prediction interval was subtracted from the observed rate for each of the 13 weeks in the 2019/2020 season to estimate the weekly excess percentages. The weekly excess percentage was multiplied by the weekly patient visits and aggregated to estimate the excess cases.

**Figure 4:**
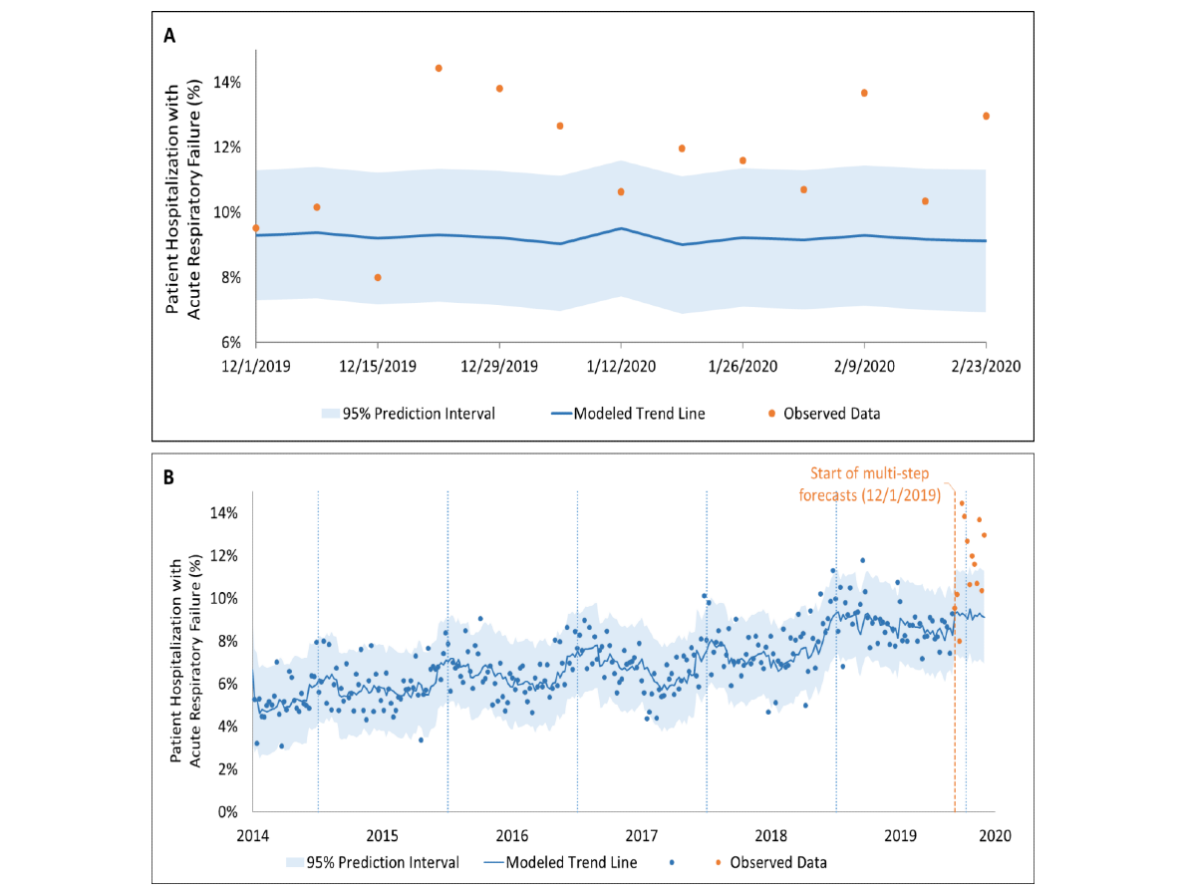
Time-series analyses of hospitalizations for acute respiratory failure. (A) Forecast, with 95% prediction intervals, of expected rates of patients hospitalized with acute respiratory failure (based on time-series analyses of the previous 5 years), with observed data for each week shown for December 1, 2019, to February 29, 2020. (B) Time-series analysis of hospitalization data.

To visualize the data, the local regression (LOESS) technique was used to smooth daily data with a smoothing span of 20% (see Figure 4 in Multimedia Appendix 1 for scatter plots of daily data) [11], with analyses performed in R (R Foundation for Statistical Computing) [12] and figures generated in Microsoft Excel (Microsoft Corp).

Two sensitivity analyses were performed. First, patient insurance status was considered to see if variation in insurance could explain trends over time in the outpatient and emergency department data; the analysis was repeated using only those outpatient clinics that existed for all years of the study period to ensure that changing case mix did not confound our results. Second, patients hospitalized with a broader set of respiratory illnesses were investigated: patients hospitalized with ICD codes used in a study of respiratory tract illnesses associated with influenza [13], and those with any pneumonia (see Table 7 in Multimedia Appendix 1 for a list of ICD codes). A SARIMA(2,0,2)x(1,0,1)_52_ model was used to analyze the inpatient data for “any respiratory tract disease,” and a SARIMA(2,0,1)x(1,0,1)_52_ model was used to analyze the inpatient data for “pneumonia.” Institutional Review Board approval was obtained (UCLA number 20-000528).

## Results

### Outpatient Clinic Data

The data encompass 9,501,091 outpatient clinic visits, with the average number of clinic visits increasing over time (eg, there were 314,832 visits from December 1, 2014, to February 28, 2015, and 511,687 visits during the 2019/2020 winter season). The expected cyclical increase in patients presenting with reports of cough each winter is observed for all 6 years studied (Figure 1A; Table 1; Figure 1A in Multimedia Appendix 1). The percentage of patients presenting for complaint of a cough was within the prediction intervals in early and mid-December 2019. Starting the week of December 22, 2019, the data exceeded the 95% prediction interval and consistently exceeded the 95% prediction interval each week through the end of February 2020 (Figure 2).

The estimated number of total excess visits for cough over the three winter months of 2019-2020 was 739 (1.6/1000 visits) when compared with the highest historical monthly value and 1047 (2.0/1000 visits) when compared with the average monthly value for all 5 years of historical controls. There were 168 excess visits above the 95% prediction interval forecast according to the time-series analysis (Table 2; Table 8 in Multimedia Appendix 1).

**Table 1:** Outpatient and emergency department visits for cough and hospitalizations for acute respiratory failure by years of winter season (December to February).

Calendar year	Outpatient			Emergency department					Hospitalization				
	Total	Cough	Cough		Total	Cough		Cough		Total	Acute respiratory failure		
	Outpatient visits	Number of patients (%)	Cases per 1000	ED^a^ visits		Number of patients (%)		Cases per 1000	Hospitalizations		Number of patients (%)		Cases per 1000
2014-2015	314,832	929 (0.30)	3.0	24,127		853 (3.54)		35.4	11,016		680 (6.17)		61.7
2015-2016	391,089	1499 (0.38)	3.8	25,977		1134 (4.37)		43.7	10,925		760 (6.96)		69.6
2016-2017	405,620	1671 (0.41)	4.1	25,505		1072 (4.20)		42.0	10,831		827 (7.64)		76.4
2017-2018	425,686	1670 (0.39)	3.9	27,022		1429 (5.29)		52.9	10,640		830 (7.80)		78.0
2018-2019	446,673	1635 (0.37)	3.7	25,555		1263 (4.94)		49.4	10,646		996 (9.36)		93.6
2019-2020	511,687	2938 (0.57)	5.7	26,748		1708 (6.39)		63.9	9903		1138 (11.49)		114.9

^a^ED: emergency department.

**Table 2:** Estimated excess cases during the 2019/2020 winter season via different methods.

Estimated excesses	Outpatient (cough)	Emergency department (cough)	Hospitalization (acute respiratory failure)
Estimated excess cases per 1000 visits^a^	1.6, 2.0	11.0, 19.2	21.4, 39.1
Estimated excess cases^a^	739, 1047	229, 514	210, 387
Total number of weeks the observed data was above the 95% prediction interval	10/13 (100% of weeks after December 22, 2019)	6/13 (60% of weeks after December 22, 2019)	7/13 (70% of weeks after December 22, 2019)
Excess cases above the 95% prediction interval^b^	168	56	18

^a^Two methods were used to estimate the excess cases per 1000 visits and excess cases in the 2019/2020 winter season (December 2019, January 2020, and February 2020) compared to previous seasons. First, for a conservative estimate of excess cases per 1000 visits, using percentages we subtracted the maximum of the five previous seasons with the current season. Second, we subtracted the average of the five previous seasons from the current season. For both methods, we multiplied the excess percentage by the total number of patient visits in the current season to estimate the excess cases.

^b^For the third method, we subtracted the upper limit of the time-series 95% prediction interval from the observed rate for each of the 13 weeks in the current season to estimate the weekly excess percentages (data not shown in table). We multiplied the weekly excess percentage by the weekly patient visits and aggregated these numbers to estimate the excess cases.

### Emergency Department Visits

The emergency department data encompass 574,813 visits from July 1, 2014, to February 29, 2020, with an average of 25,822 visits per winter season. Similar to outpatient visits for cough, seasonal variation in the proportion of emergency department visits for cough was observed (Figure 1B, Table 1). An excess above the time-series 95% prediction interval was noted starting December 22, 2019; in total, 6 of the 10 weeks exceeded the 95% prediction interval (Figure 3). The estimated number of total excess in patient visits to the emergency departments for cough over the 2019/2020 winter season using the three methods was 229 (11.0/1000 visits), 514 (19.2/1000 visits), and 56, respectively (Table 2; Table 9 in Multimedia Appendix 1).

### Hospitalized Patients

There were 247,774 patients admitted to the three hospitals included in this study from July 1, 2014, to February 29, 2020, with an average of 10,660 admissions each winter season. The percentage of patients with a discharge diagnosis of acute respiratory failure were higher in December 2019, January 2020, and February 2020 when compared with all 5 historical control years (Figure 1C, Table 1). The observed percentage of patients who had acute respiratory failure during the subsequent hospitalization exceeded the time-series 95% prediction interval for patients admitted starting the week of December 22, 2019; in total, 7 of the 10 weeks of observed data were above the 95% prediction interval (Figure 4). Using the three prediction methods, the estimated excess numbers of patients hospitalized with acute respiratory failure were 210 (21.4/1000 visits), 387 (39.1/1000 visits), and 18, respectively (Table 2; Table 10 in Multimedia Appendix 1).

### Sensitivity Analyses

The above findings were qualitatively unchanged when clinic and emergency department visits were analyzed by insurance class and clinic visits were restricted to clinics that treated patients in all years of the study period (Figures 5-7 in Multimedia Appendix 1). The percentage of patients with a discharge diagnosis of any respiratory tract disease or of any pneumonia were also higher in December 2019, January 2020, and February 2020 when compared with prior years (Table 11 in Multimedia Appendix 1).

## Discussion

A significantly higher number of patients presented to outpatient clinics and emergency departments in this health system with a complaint of cough starting the last week of December 2019 and continuing through January and February 2020. These findings translate into hundreds of additional patients seeking outpatient medical attention in this health system for the respiratory symptom of cough during the 2019/2020 winter season. A significant excess in the number of patients hospitalized with acute respiratory failure during this same time period was also noted. It is possible that some of this excess represents early COVID-19 disease before clinical recognition and testing, information that may help epidemiologists better understand the spread of this pandemic. If only some of these excess visits are due to COVID-19, this could still represent community spread of SARS-CoV-2 during that time because a substantial proportion of individuals infected have no symptoms or mild symptoms and do not seek medical care [14,15], making cryptic spread of the disease within a community likely.

EHRs are widely adopted but have not been used to their fullest potential to deliver high-value care [16-18]. This work demonstrates the potential of using EHR data for symptom or disease surveillance. A strength of this study is the use of raw EHR data that are already collected in most health care systems to determine whether patients were presenting with reports of “cough” at excess numbers in the months before the first known case of COVID-19 in this health system. In addition, this study considers data from three separate locations of patient care contained in the EHR: the outpatient setting, the emergency department, and the hospital. For many diseases, data from the outpatient setting can provide an early warning to emergency departments and hospital intensive care units of what is to come. By leveraging time-series analysis to calculate prediction intervals of expected patients, outlier numbers of patient visits can be quickly identified.

While asymptomatic transmission and community spread of COVID-19 are possible explanations for the observed excess patient encounters, other reasons and limitations need to be considered. The study was performed in a single health system and we only searched for the word “cough.” Although the term “cough” is possibly more specific to COVID-19 than other symptoms such as “fever” or “aches,” this search method has imperfect specificity and sensitivity as it does not include the full spectrum of COVID-19 symptoms [19-25]. The health system patient mix could have changed over the study period, but sensitivity analyses did not find evidence that this affected the findings. It is possible that the findings are due to lung injury from e-cigarettes (vaping), but this explanation is doubtful because the Centers for Disease Control and Prevention reported a continued decline after September 2019 [26].

Another limitation is not knowing for certain whether and what percentage of excess patient visits were due to influenza. An increase in influenza-positive test results and emergency department visits for influenza-like illnesses was noted in Los Angeles County and the United States during the 2019/2020 winter season when compared with prior years [27,28]. The incidence of influenza-like illness symptoms in the United States peaked earlier in 2019 when compared with previous years [28], with the 2019/2020 season peak at a similar level as the 2017/2018 season peak. This suggests that some of the observed incidence was due to influenza rather than COVID-19. However, the analysis shows an excess of patients above the forecast 95% prediction interval based on five previous seasons.

It is plausible that some of the excess visits might be due to SARS-CoV-2 as studies of rapid sentinel surveillance and genome sequencing suggest community transmission of SARS-CoV-2 much earlier than initially thought. Studies in the United States and France found evidence for cryptic spread of the virus as early as December 2019 or January 2020, before community surveillance was actively implemented [29-32]. Especially early in outbreaks, existing methods for case identification may not capture incident infections; thus, novel and complementary methods using EHR data such as those reported here may play an important role.

Heightened media attention regarding the coronavirus pandemic could influence patients to seek medical care for cough-related concerns and should be considered [33]. Such data can complement these EHR data approaches. Using data from Google Trends [34], the popularity of “cough” as a search term in the United States was slightly higher from late December 2019 through January 2020 when compared with the average national popularity in the previous 4 years (Figure 8 in Multimedia Appendix 1). Popularity of searches for “cough” increased substantially in mid-March, corresponding to the sharp increase in “coronavirus” Google news searches. Although there was almost no mention of a COVID-19–type illness by United States media in December 2019 and little mention in January 2020, substantial media attention was present in February 2020. Therefore, this limitation is pronounced for February 2020, but less concerning for the earlier months.

In summary, health system analytics combined with EHR data can be harnessed to quickly identify changes in underlying patient populations. This study identified a significant excess of patients with COVID-19–like presentations starting the last week of December 2019 and continuing through February 2020, a time period before the availability of testing or providers considered clinical diagnoses of COVID-19. A unique feature of this study is the evaluation of three different stages of health care settings, which expands surveillance beyond just reporting the number of patients in emergency departments or hospital intensive care unit beds to include consideration of 9.5 million outpatient clinic records. The Centers for Disease Control and Prevention Outpatient Influenza-like Illness Surveillance Network (ILINet) currently monitors patients presenting with fever and a cough and/or a sore throat in the United States [35]. Data from the outpatient clinic setting is usually a harbinger of what is to come for hospital emergency departments and intensive care units.

Harnessing larger electronic health data systems to monitor outpatient visits for the growing and diverse set of symptoms associated with COVID-19 should be considered [21-25].

This SARS-CoV-2 pandemic highlights the urgent need to support the development of agile health care analytics that enable real-time symptom and disease surveillance [36]. Lessons learned from this pandemic will hopefully lead to better preparation and the ability to quickly provide warnings and track the next pandemic.

## Appendix

Multimedia Appendix 1 All supplementary figures and tables.

## Abbreviations

EHR: electronic health record
ICD: International Classification of Diseases
SARIMA: seasonal autoregressive integrated moving average
SCAN: smallest canonical correlation
WHO: World Health Organization
