# Supplementary material for: Excess Patient Visits for Cough and Pulmonary Disease at a Large US Health System in the Months Prior to the COVID-19 Pandemic: Time-Series Analysis
Source: J Med Internet Res. 2020 Sep 10;22(9):e21562. doi: 10.2196/21562 (PMC7485935; doi:10.2196/21562)
Supplement: Multimedia Appendix 1 [file jmir_v22i9e21562_app1.docx]

**ELECTRONIC APPENDIX**

EXCESS PATIENT VISITS FOR COUGH AND PULMONARY DISEASE

AT A LARGE U.S. HEALTH SYSTEM

IN THE MONTHS PRIOR TO THE COVID-19 PANDEMIC: A TIME-SERIES ANALYSIS

(JOANN G. ELMORE, MD, MPH, ET. AL.)

TABLE OF CONTENTS

Methods anD results for time-series analyses 3

Outpatient Data (etable 1) 4

Outpatient sensitivity analysis (etable 2; efigure 1a-1b) 5

Emergency department data (etable 3) 7

Hospitalization – any respiratory tract disease (etable 4; efigure 2a-2b) 8

Hospitalization – any pneumonia (etable 5; efigure 3a-3b) 10

Hospitalization – acute Respiratory failure (etable 6) 12

etable 7: List of Icd codes 13

etable 8: outpatient visit data 19

etable 9: Emergency department data 20

etable 10: hospitalization data 21

etable 11: hospitalizations for any repiratory tract disease or pneumonia 23

eFigure 4: scatter plots daily Data and smooth lines 24

efigure 5: outpatient visit data by insurance status 29

efigure 6: outpatient visit dAtA from clinics consistently operated since 2014 30

efigure 7: Emergency department visit data by insurance status 31

efigure 8: Google search trends 32

# Methods and Results for Time-Series Analyses

For formal time-series analyses, we used seasonal autoregressive integrated moving average (SARIMA) models on the weekly data. These models take into account seasonal effects (Box and Tiao, 1975, Shumway and Stoffer, 2017).

We used maximum likelihood to estimate model parameters using data from July 1, 2014 to November 30, 2019. We then forecast the values expected for December 1, 2019 through February 29, 2020 using the estimated model and evaluated whether the observed data were unusual compared to these forecasts. We used the Time Series Forecasting analysis ARIMA procedure of SAS/ETS software to fit and forecast the ARIMA models (Brocklebank, 2003). On the following pages we provide summary tables and graphs of the model fits, showing forecast on the far right side of the graphs, followed by zoomed-in graphs of the forecast time period with observed data shown for each weekly interval during the winter season 2019/2020.

**References:**

Box, G. E. P., and Tiao, G. C. (1975). “Intervention Analysis with Applications to Economic and Environmental Problems.” Journal of the American Statistical Association 70:70–79.

Brocklebank, J. C., and Dickey, D. A. (2003). SAS for Forecasting Time Series. 2nd ed. Cary, NC: SAS Institute Inc.

Shumway, Robert H., and David S. Stoffer. Time series analysis and its applications: with R examples. Springer, 2017.”

## Outpatient Data

### Model

The outpatient data time series, aggregated by week, is shown in **Figure 2** of the paper. For this time series, after model selection procedures, we chose the following $SARIMA(1,0,1)\times\left( 1,0,1 \right)_{52}$ model:

$$\left( 1-\phi_{1}B \right)\left( 1-\phi_{2}B^{52} \right)\left[ X_{t}-\left( \beta_{0}+\beta_{1}Z_{t} \right) \right]=\left( 1-\theta_{1}B \right)\left( 1-\theta_{2}B^{52} \right)W_{t}$$

where:

$X_{t}$ is the percentage of visits in week $t$ for which cough was a recorded symptom

$B$ is the backshift operator, $BX_{t}=X_{t-1}$

$W_{t}\sim\mathrm{wn}(0,\sigma_{w}^{2})$ is a white noise process of uncorrelated random variables with mean 0 and variance $\sigma_{w}^{2}$

$Z_{t}$ is an indicator variable for the months of December-February

The model parameter estimates are shown in eTable 1.

### Results

**eTable 1**: ARIMA model parameter estimates for outpatient visits data

| Parameter | Estimate | Standard  Error | t Value | *P* Value |
| --- | --- | --- | --- | --- |
| $\boldsymbol{\beta}_{\boldsymbol{0}}$ | 0.00257 | 0.000316 | 8.13 | <.001 |
| $\boldsymbol{\beta}_{\boldsymbol{1}}$ | 0.000466 | 0.0002509 | 1.86 | .06 |
| $\boldsymbol{\theta}_{\boldsymbol{1}}$ | 0.46909 | 0.07250 | 6.47 | <.001 |
| $\boldsymbol{\theta}_{\boldsymbol{2}}$ | 0.60713 | 0.13199 | 4.60 | <.001 |
| $\boldsymbol{\phi}_{\boldsymbol{1}}$ | 0.88854 | 0.03889 | 22.85 | <.001 |
| $\boldsymbol{\phi}_{\boldsymbol{2}}$ | 0.91870 | 0.05763 | 15.94 | <.001 |

### Conclusions

The percent of patients presenting for complaint of a cough was within the prediction intervals in early and mid-December and then exceeded the 95% prediction intervals starting during the week of December 22, 2019 and was consistently above the 95% prediction interval each week through the end of February 2020

## Outpatient Sensitivity Analysis

In **Figure 2** in the paper, there are several outliers near the last weeks of December for several years. We were concerned that these outliers were due to drop-offs in the rates of visits for other causes, causing the denominator of the analyzed percentages to decrease, rather than increases in the absolute rates of visits for cough. Therefore, a sensitivity analysis was performed. We linearly interpolated the all-cause visit counts for week 51 and 52 of each year, and refit the model using these adjusted denominators.

### Model

The adjusted outpatient data time series, aggregated by week, is shown in eFigure 1A and 1B. For this time series, after model selection procedures, we chose the following $SARIMA(3,0,1)\times\left( 1,0,1 \right)_{52}$ model:

$$\left( 1-\phi_{1,1}B-\phi_{1,2}B^{2}-\phi_{1,3}B^{3} \right)\left( 1-\phi_{2,1}B^{52} \right)\left[ X_{t}-\left( \beta_{0}+\beta_{1}Z_{t} \right) \right]=\left( 1-\theta_{1}B \right)\left( 1-\theta_{2}B^{52} \right)W_{t}$$

where:

$X_{t}$ is the percentage of visits in week $t$ for which cough was a recorded symptom

$B$ is the backshift operator, $BX_{t}=X_{t-1}$

$W_{t}\sim\mathrm{wn}(0,\sigma_{w}^{2})$ is a white noise process of uncorrelated random variables with mean 0 and variance $\sigma_{w}^{2}$

$Z_{t}$ is an indicator variable for the months of December-February

The model parameter estimates are shown in eTable 2.

### Results

**eTable 2:** model parameter estimates for outpatient cough data, with all-cause visits interpolated for weeks 51 and 52 of each year.

| Parameter | Estimate | Standard  Error | t Value | *P* Value |
| --- | --- | --- | --- | --- |
| $\boldsymbol{\beta}_{\boldsymbol{0}}$ | 0.00253 | 0.00029 | 8.73 | <.001 |
| $\boldsymbol{\beta}_{\boldsymbol{1}}$ | 0.0004 | 0.00019 | 2.07 | .04 |
| $\boldsymbol{\theta}_{\boldsymbol{1}}$ | 0.53895 | 0.33743 | 1.6 | .11 |
| $\boldsymbol{\theta}_{\boldsymbol{2}}$ | 0.52851 | 0.22831 | 2.31 | .02 |
| $\boldsymbol{\phi}_{\boldsymbol{1,1}}$ | 1.06646 | 0.34113 | 3.13 | .002 |
| $\boldsymbol{\phi}_{\boldsymbol{1,2}}$ | -0.1149 | 0.19151 | -0.6 | .55 |
| $\boldsymbol{\phi}_{\boldsymbol{1,3}}$ | -0.0196 | 0.12353 | -0.16 | .87 |
| $\boldsymbol{\phi}_{\boldsymbol{2,1}}$ | 0.77192 | 0.18282 | 4.22 | <.001 |

**
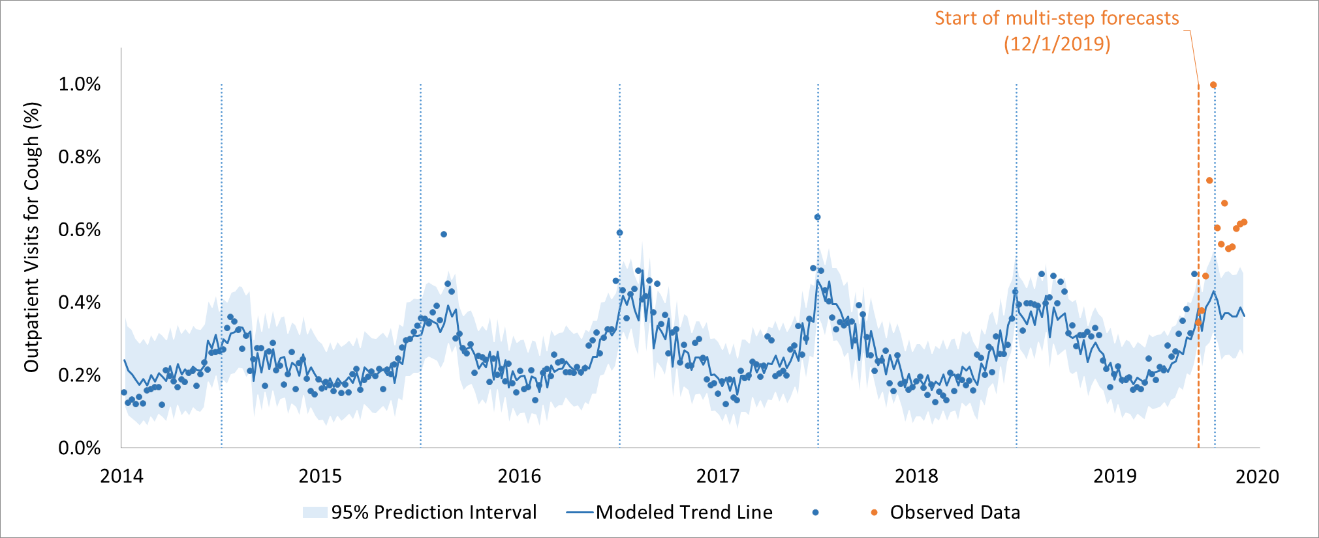
**

**eFigure 1A:** Percentages of outpatient visits with cough recorded as a symptom, aggregated by week, and fitted model (forecasts and 95% forecast intervals), with all-cause visits interpolated for weeks 51 and 52 of each year.

**
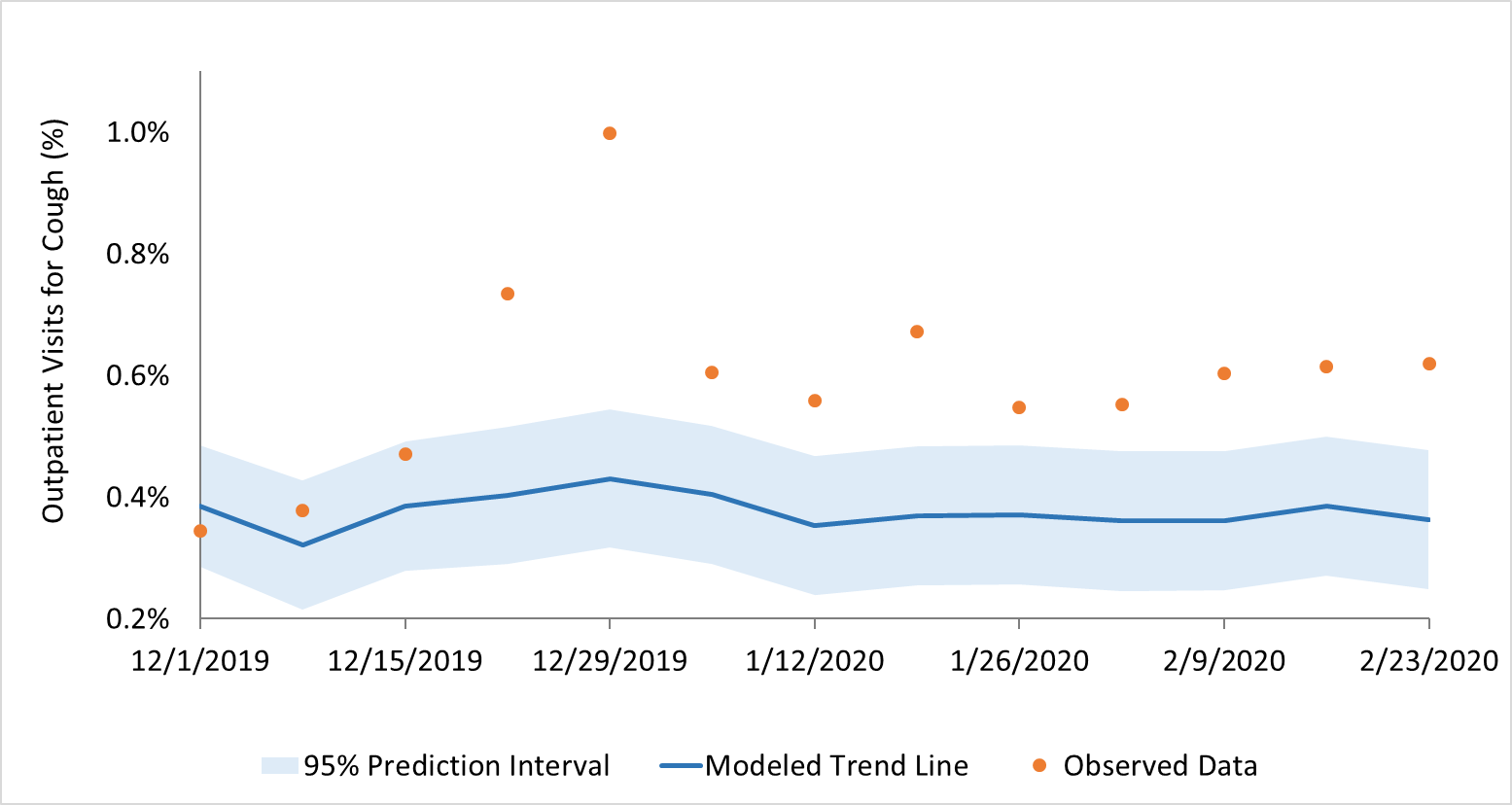
**

**eFigure 1B:** Forecasts and observed percentages of outpatient visits for cough, Dec 2019-Feb 2020, with all-cause visits interpolated for weeks 51 and 52 of each year.

### Conclusions

The findings of this sensitivity analysis using data interpolated for weeks 51 and 52 of each year were similar to the primary analysis.

## Emergency Department Data

### Model

The emergency department data time series, aggregated by week, is shown in **Figure 3** in the paper. For this time series, after model selection procedures, we chose the following $SARIMA(1,0,3)\times\left( 1,0,1 \right)_{52}$ model:

$$\left( 1-\phi_{1}B \right)\left( 1-\phi_{2}B^{52} \right)\left[ X_{t}-\left( \beta_{0}+\beta_{1}Z_{t} \right) \right]=\left( 1-\theta_{1,1}B-\theta_{1,2}B^{2}-\theta_{1,3}B^{3} \right)\left( 1-\theta_{2,1}B^{52} \right)W_{t}$$

where:

$X_{t}$ is the percentage of visits in week $t$ for which cough was a recorded symptom

$B$ is the backshift operator, $BX_{t}=X_{t-1}$

$W_{t}\sim\mathrm{wn}(0,\sigma_{w}^{2})$ is a white noise process of uncorrelated random variables with mean 0 and variance $\sigma_{w}^{2}$

$Z_{t}$ is an indicator variable for the months of December-February

The model parameter estimates are shown in eTable 3.

### Results

**eTable 3:** ARIMA model parameter estimates for Emergency Department visits

| Parameter | Estimate | Standard Error | t Value | *P* Value |
| --- | --- | --- | --- | --- |
| $\boldsymbol{\beta}_{\boldsymbol{0}}$ | 0.02971 | 0.0028649 | 10.37 | <.001 |
| $\boldsymbol{\beta}_{\boldsymbol{1}}$ | 0.0052941 | 0.0023594 | 2.24 | .03 |
| $\boldsymbol{\theta}_{\boldsymbol{1,1}}$ | 0.36598 | 0.07140 | 5.13 | <.001 |
| $\boldsymbol{\theta}_{\boldsymbol{1,2}}$ | 0.09157 | 0.06625 | 1.38 | .17 |
| $\boldsymbol{\theta}_{\boldsymbol{1,3}}$ | -0.16015 | 0.06433 | -2.49 | .01 |
| $\boldsymbol{\theta}_{\boldsymbol{2,1}}$ | 0.20481 | 0.23757 | 0.86 | .39 |
| $\boldsymbol{\phi}_{\boldsymbol{1}}$ | 0.87305 | 0.04499 | 19.40 | <.001 |
| $\boldsymbol{\phi}_{\boldsymbol{2}}$ | 0.52597 | 0.21171 | 2.48 | .02 |

### Conclusions

An increase above the 95% prediction interval was noted starting December 22, 2019; 7 of the 10 weeks exceeded the 95% prediction interval.

## Hospitalization – Any respiratory tract disease

### Model

The inpatient data time series for “any respiratory tract disease”, aggregated by week, is shown in **eFigure 2.** For this time series, after model selection procedures, we chose the following $SARIMA(2,0,2)\times\left( 1,0,1 \right)_{52}$ model:

$$\left( 1-\phi_{1,1}B-\phi_{1,2}B^{2} \right)\left( 1-\phi_{2,1}B^{52} \right)\left[ X_{t}-\left( \beta_{0}+\beta_{1}Z_{t} \right) \right]=\left( 1-\theta_{1,1}B-\theta_{1,2}B^{2} \right)\left( 1-\theta_{2,1}B^{52} \right)W_{t}$$

where:

$X_{t}$ is the percentage of visits in week $t$ for which cough was a recorded symptom

$B$ is the backshift operator, $BX_{t}=X_{t-1}$

$W_{t}\sim\mathrm{wn}(0,\sigma_{w}^{2})$ is a white noise process of uncorrelated random variables with mean 0 and variance $\sigma_{w}^{2}$

$Z_{t}$ is an indicator variable for the months of December-February

The model parameter estimates are shown in eTable 4.

### Results

**eTable 4:** ARIMA model parameter estimates for hospitalizations for any respiratory tract diseases

| Parameter | Estimate | Standard  Error | t Value | *P* Value |
| --- | --- | --- | --- | --- |
| $\boldsymbol{\beta}_{\boldsymbol{0}}$ | 0.12372 | 0.0056890 | 21.75 | <.001 |
| $\boldsymbol{\beta}_{\boldsymbol{1}}$ | 0.02162 | 0.0050951 | 4.24 | <.001 |
| $\boldsymbol{\theta}_{\boldsymbol{1,1}}$ | 0.6207 | 0.33257 | 1.87 | .06 |
| $\boldsymbol{\theta}_{\boldsymbol{1,2}}$ | 0.14905 | 0.21089 | 0.71 | .48 |
| $\boldsymbol{\theta}_{\boldsymbol{2,1}}$ | 0.61928 | 0.29875 | 2.07 | .04 |
| $\boldsymbol{\phi}_{\boldsymbol{1,1}}$ | 1.024 | 0.33346 | 3.07 | .002 |
| $\boldsymbol{\phi}_{\boldsymbol{1,2}}$ | -0.0789 | 0.29804 | -0.26 | .79 |
| $\boldsymbol{\phi}_{\boldsymbol{2,1}}$ | 0.79422 | 0.24685 | 3.22 | .001 |

**eFigure 2A**: Percentages of hospitalization with any respiratory tract disease, aggregated by week, and fitted model (forecasts and 95% forecast intervals).

**eFigure 2B:** Forecasts and observed percentages of hospitalization with any respiratory tract disease, Dec 2019-Feb 2020.

### Conclusions

### The findings of our time-series analysis with using the ICD codes for any respiratory tract disease were similar to our primary analysis.

## Hospitalization – Any pneumonia

### Model

The hospitalization pneumonia data time series, aggregated by week, is shown in **eFigure 3A** and **3B.** For this time series, after model selection procedures, we chose the following $SARIMA(2,0,1)\times\left( 1,0,1 \right)_{52}$ model:

$$\left( 1-\phi_{1,1}B-\phi_{1,2}B^{2} \right)\left( 1-\phi_{2,1}B^{52} \right)\left[ X_{t}-\left( \beta_{0}+\beta_{1}Z_{t} \right) \right]=\left( 1-\theta_{1}B \right)\left( 1-\theta_{2}B^{52} \right)W_{t}$$

where:

$X_{t}$ is the percentage of visits in week $t$ for which cough was a recorded symptom

$B$ is the backshift operator, $BX_{t}=X_{t-1}$

$W_{t}\sim\mathrm{wn}(0,\sigma_{w}^{2})$ is a white noise process of uncorrelated random variables with mean 0 and variance $\sigma_{w}^{2}$

$Z_{t}$ is an indicator variable for the months of December-February

The model parameter estimates are shown in eTable 5.

### Results

**eTable 5: ARIMA model parameter estimates for hospitalizations for pneumonia**

| Parameter | Estimate | Standard  Error | t Value | *P* Value |
| --- | --- | --- | --- | --- |
| $\boldsymbol{\beta}_{\boldsymbol{0}}$ | 0.07343 | 0.0022809 | 32.19 | <.001 |
| $\boldsymbol{\beta}_{\boldsymbol{1}}$ | 0.0196 | 0.0036158 | 5.42 | <.001 |
| $\boldsymbol{\theta}_{\boldsymbol{1,1}}$ | 0.77193 | 0.25105 | 3.07 | .002 |
| $\boldsymbol{\theta}_{\boldsymbol{1,2}}$ | 0.91306 | 0.47233 | 1.93 | .05 |
| $\boldsymbol{\theta}_{\boldsymbol{2,1}}$ | 1.12595 | 0.26482 | 4.25 | <.001 |
| $\boldsymbol{\phi}_{\boldsymbol{1}}$ | -0.23225 | 0.13577 | -1.71 | .09 |
| $\boldsymbol{\phi}_{\boldsymbol{2}}$ | 0.98397 | 0.16437 | 5.99 | <.001 |

**eFigure 3A:** Percentages of hospitalization with any pneumonia, aggregated by week, and fitted model (forecasts and 95% forecast intervals).

**eFigure3B:** Forecasts and observed percentages of hospitalization with any pneumonia, Dec 2019-Feb 2020.

### Conclusions

We again note an excess of patients hospitalized with pneumonia starting the end of December, with 5 of the 10 weeks above the 95% prediction interval.

## Hospitalization – Acute Respiratory Failure

### Model

The “Hospitalization – Acute Respiratory Failure” data time series, aggregated by week, is shown in **Figure 4** in the paper. For this time series, after model selection procedures, we chose the following $SARIMA(1,0,1)\times\left( 1,0,1 \right)_{52}$ model:

$$\left( 1-\phi_{1}B \right)\left( 1-\phi_{2}B^{52} \right)\left[ X_{t}-\left( \beta_{0}+\beta_{1}Z_{t} \right) \right]=\left( 1-\theta_{1}B \right)\left( 1-\theta_{2}B^{52} \right)W_{t}$$

where:

$X_{t}$ is the percentage of visits in week $t$ for which cough was a recorded symptom

$B$ is the backshift operator, $BX_{t}=X_{t-1}$

$W_{t}\sim\mathrm{wn}(0,\sigma_{w}^{2})$ is a white noise process of uncorrelated random variables with mean 0 and variance $\sigma_{w}^{2}$

$Z_{t}$ is an indicator variable for the months of December-February

The model parameter estimates are shown in eTable 6.

### Results

**eTable 6:** ARIMA model parameter estimates for hospitalizations for acute respiratory failure

| Parameter | Estimate | Standard  Error | t Value | *P* Value |
| --- | --- | --- | --- | --- |
| $\boldsymbol{\beta}_{\boldsymbol{0}}$ | 0.0668 | 0.01398 | 4.78 | <.001 |
| $\boldsymbol{\beta}_{\boldsymbol{1}}$ | 0.0078561 | 0.0019187 | 4.09 | <.001 |
| $\boldsymbol{\theta}_{\boldsymbol{1}}$ | 0.8633 | 0.03556 | 24.28 | <.001 |
| $\boldsymbol{\theta}_{\boldsymbol{2}}$ | -0.74722 | 0.46799 | -1.6 | .11 |
| $\boldsymbol{\phi}_{\boldsymbol{1}}$ | 0.99487 | 0.0069182 | 143.81 | <.001 |
| $\boldsymbol{\phi}_{\boldsymbol{2}}$ | -0.63052 | 0.47787 | -1.32 | .19 |

### Conclusions

The observed percent of patients who had acute respiratory failure during the subsequent hospitalization exceed the 95% prediction interval for patients admitted starting the week of December 22, 2019; 7 of the 10 weeks of observed data were above the 95% prediction interval. These figures are shown in the paper Figure 4A, B.

**eAppendix Table 7.** List of ICD codes searched for A. Respiratory tract diagnoses , B. Pneumonia, C. Acute respiratory failure.

**Set A. Respiratory tract diagnoses (From Chow et al, 2020)**

| **ICD-9** | **ICD-10** | **dx_name** |
| --- | --- | --- |
| **Acute Respiratory Distress Syndrome** | | |
| 518.82 | J80 | Acute respiratory distress syndrome |
| **Acute Upper Respiratory Infections** | | |
| 460-465 | J00-J06 | Acute upper respiratory infections |
| **Asthma Exacerbation** | | |
|  | J45.21 | Mild intermittent asthma with acute exacerbation |
|  | J45.22 | Mild intermittent asthma with status asthmaticus |
|  | J45.31 | Mild persistent asthma with acute exacerbation |
|  | J45.32 | Mild persistent asthma with status asthmaticus |
|  | J45.41 | Moderate persistent asthma with acute exacerbation |
|  | J45.42 | Moderate persistent asthma with status asthmaticus |
|  | J45.51 | Severe persistent asthma with acute exacerbation |
|  | J45.52 | Severe persistent asthma with status asthmaticus |
| 493.92 | J45.901 | Unspecified asthma with acute exacerbation |
| 493.91 | J45.902 | Unspecified asthma with status asthmaticus |
| 493.01 |  | Extrinsic asthma with status asthmaticus |
| 493.02 |  | Extrinsic asthma with acute exacerbation |
| 493.11 |  | Intrinsic asthma with status asthmaticus |
| 493.12 |  | Intrinsic asthma with acute exacerbation |
| 493.21 |  | Chronic obstructive asthma with status asthmaticus |
| 493.22 |  | Chronic obstructive asthma with acute exacerbation |
|  |  | Chronic Obstructive Pulmonary Disease Exacerbation |
| **Chronic Obstructive Pulmonary Disease Exacerbation** | | |
| 491.21 | J44.1 | COPD with acute exacerbation |
| **Mediastinitis** | | |
| 519.2 | J98.51 | Mediastinitis Mediastinitis |
| **Other Acute Lower Respiratory Tract Disease** | | |
| 466.0 | J20 | Acute bronchitis |
| 466.1 | J21 | Acute bronchiolitis |
| 519.8 | J22 | Unspecified acute lower respiratory infection |
| 491.22 | J44.0 | COPD with acute lower respiratory infection |
|  | J47.1 | Bronchiectasis with acute lower respiratory infection |
| 494.1 | J47.9 | Bronchiectasis with acute exacerbation |
| 513 |  | Abscess of lung and mediastinum |
|  | J85.0 | Gangrene and necrosis of lung |
| 513.0 | J85.2 | Abscess of lung without pneumonia |
| 513.1 | J85.3 | Abscess of mediastinum |
| 510 | J86 | Pyothorax |
| 510.0 | J86.0 | Pyothorax with fistula |
| 510.9 | J86.9 | Pyothorax without fistula |
| **Pneumonia** | | |
| 480 | J12 | Viral pneumonia |
| 481 | J13 | Streptococcus pneumoniae pneumonia |
| 482.2 | J14 | Hemophilus influenzae pneumonia |
| 482 | J15 | Other bacterial pneumonia |
| 483 | J16 | Pneumonia due to other specified organism |
| 484 | J17 | Pneumonia in infectious diseases classified elsewhere |
| 486 | J18 | Pneumonia, unspecified organism |
| 485 |  | Bronchopneumonia, organism unspecified |
| 513.0 | J85.1 | Abscess of lung with pneumonia |
| 488.81 | J09.X1 | Influenza due to identified novel influenza A virus with pneumonia |
| 488.01 | J10.0 | Influenza due to other identified influenza virus with pneumonia |
|  | J11.0 | Influenza due to unidentified influenza virus with pneumonia |
| 488.11 |  | Influenza due to identified 2009 H1N1 influenza virus with pneumonia |
| 487.0 |  | Influenza with pneumonia |
| **Pneumothorax** | | |
| 512 | J93 | Pneumothorax and air leak |
| **Pulmonary Collapse** | | |
| 518.0 | J98.11 | Atelectasis |
| 518.0 | J98.19 | Other pulmonary collapse |
| **Respiratory Failure** | | |
| 518.81 | J96.0 | Acute respiratory failure |
| 518.84 | J96.2 | Acute and chronic respiratory failure |
| 799.1 | R09.2 | Respiratory arrest |
| **Influenza with Other Respiratory Manifestations** | | |
|  | J09.X2 | Influenza due to identified novel influenza A virus with other respiratory manifestations |
|  | J10.1 | Influenza due to other identified influenza virus with other respiratory manifestations |
|  | J11.1 | Influenza due to unidentified influenza virus with other respiratory manifestations |
| 487.1 |  | Influenza with other respiratory manifestations |
| 488.02 |  | Influenza due to identified avian influenza with other respiratory manifestations |
| 488.12 |  | Influenza due to identified 2009 H1N1 influenza virus with other respiratory manifestations |
| 488.82 |  | Influenza due to identified novel influenza A virus with other respiratory manifestations |

**Set B: Pneumonia**

| **ICD-10 code** | **Description** |
| --- | --- |
| B97.29 | Other coronavirus as cause of disease elsewhere classed |
| J09.X1 | Influenza due to identified novel influenza A virus with pneumonia |
| J09.X2 | Influenza due to identified novel influenza A virus with other respiratory manifestations |
| J09.X3 | Influenza due to identified novel influenza A virus with gastrointestinal manifestations |
| J09.X9 | Influenza due to identified novel influenza A virus with other manifestations |
| J10.01 | Influenza due to other identified influenza virus with the same other identified influenza virus pneumonia |
| J10.08 | Influenza due to other identified influenza virus with other specified pneumonia |
| J10.1 | URI due to novel H1N1 influenza virus |
| J10.2 | Novel H1N1 influenza with involvement of gastrointestinal tract |
| J10.81 | Influenza due to other identified influenza virus with encephalopathy |
| J10.89 | Influenza due to other influenza virus with other manifestations |
| J11.1 | Influenza due to unidentified influenza virus with other respiratory manifestations |
| J11.00 | Primary influenza virus pneumonia |
| J11.08 | Influenzal bronchopneumonia |
| J11.1 | URI due to influenza A virus |
| J12.0 | Respiratory disease due to adenovirus |
| J12.1 | RSV (respiratory syncytial virus pneumonia) |
| J12.2 | Pneumonia due to parainfluenza virus |
| J12.3 | Pneumonia due to human metapneumovirus (hMPV) |
| J12.81 | SARS virus pneumonia |
| J12.89 | Pneumonitis due to herpes zoster (OTHER VIRAL PNEUMONIA NEC) |
| J12.9 | Viral pneumonitis (VIRAL PNEUMONIA UNSPECIFIED) |
| J13 | Streptococcus pneumoniae pneumonia (HCC/RAF) |
| J14 | Pneumonia, Hemophilus influenzae |
| J15.0 | Pneumonia, Klebsiella (HCC/RAF) |
| J15.1 | Pseudomonas pneumonia (HCC/RAF) |
| J15.20 | Staphylococcal pneumonia, right (HCC/RAF) |
| J15.211 | Staphylococcus aureus pneumonia (HCC/RAF) |
| J15.212 | Pneumonia of upper lobe due to methicillin resistant Staphylococcus aureus (MRSA), unspecified laterality (HCC/RAF) |
| J15.29 | Pneumonia due to other staphylococcus (HCC/RAF) |
| J15.3 | Pneumonia of upper lobe due to group B Streptococcus, unspecified laterality (HCC/RAF) |
| J15.4 | Streptococcal pneumonia (HCC/RAF) |
| J15.5 | Pneumonia of upper lobe due to Escherichia coli, unspecified laterality (HCC/RAF) |
| J15.6 | Pulmonary actinobacillosis (HCC/RAF) |
| J15.7 | Primary atypical pneumonia due to Mycoplasma pneumoniae |
| J15.8 | Pneumonia due to other specified bacteria(482.89) (HCC/RAF) |
| J15.9 | Unspecified bacterial pneumonia |
| J16.0 | Pneumonia, Chlamydia |
| J16.8 | Pneumonia due to other specified organism(483.8) |
| J17 | Rheumatic pneumonia |
| J18.0 | Terminal bronchopneumonia |
| J18.1 | Upper lobe consolidation (HCC/RAF) |
| J18.2 | Passive pneumonia |
| J18.8 | Other pneumonia, unspecified organism |
| J18.9 | Worsening pneumonia (PNEUMONIA, UNSPECIFIED ORGANISM) |
| J22 | Unspecified acute lower respiratory infection |
| J69.0 | Vomit inhalation pneumonitis (HCC/RAF) |
| J69.1 | Pneumonitis due to oils and essences (HCC/RAF) |
| J95.851 | Ventilator-associated bacterial pneumonia (HCC/RAF) |
| J10.00 | Influenza due to other identified influenza virus with unspecified type of Pneumonia |
| J84.89 | Organizing Pneumonia, Interstitial pneumonitis |
| B37.1 | Candidal pneumonia |
| J84.116 | Cryptogenic organizing pneumonia |
| B25.0 | Cytomegaloviral pneumonitis |
| J84.117 | Desquamative interstitial pneumonia |
| J85.0 | Pulmonary gangrene and necrosis |
| J85.1 | Pneumonia with lung abscess |
| B05.2 | Measles Pneumonia |
| A54.84 | Gonococcal pneumonia |
| A42.0 | Pneumonia due to actinomycosis |
| B44.9 | Pneumonia in aspergillosis |
| B01.2 | Varicella Pneumonia |
| B38.0, B38.1, B38.2 | Pulmonary codidioidomycosis |
| B39.0, B39.1, B39.2 | Pneumonia due to histoplasma (acute, chronic, and Uns) |
| A43.0 | Pneumonia due to nocardiasis |
| B59 | Pneumonia due to Pneumocystis carinii |
| A24.1 | Melioidosis pneumonia |
| B06.81 | Rubella Pneumonia |
| A02.22 | Salmonella Pneumonia |
| A01.03 | Typhoid Pneumonia |
| A37.01, A37.11, A37.81, A37.91 | Pneumonia with whooping cough of various types |
| J84.17 | Interstitial Pneumonia in diseases classified elsewhere |
| J84.2 | Lymphoid interstitial pneumonia |
| J84.113 | Idiopathic non-specific interstitial pneumonitis |

**Set C: Acute respiratory failure**

| **code** | **Description** |
| --- | --- |
| J80 | Acute respiratory distress syndrome (ARDS) |
| J96.00 | acute respiratory failure (HCC/RAF) |
| J96.01 | acute hypoxic respiratory failure, unspecified (HCC/RAF) |
| J96.02 | acute hypercapnic respiratory failure (HCC/RAF) |
| J96.20 | Respiratory failure, acute-on-chronic (HCC/RAF) |
| J96.21 | Acute on chronic respiratory failure with hypoxia and hypercapnia (HCC/RAF) |
| J96.22 | Acute on chronic respiratory failure with hypoxia and hypercapnia (HCC/RAF) |
| J96.90 | Respiratory failure, unspecified, not specified whether with hypoxia or hypercapnia |
| J96.91 | (above), but with hypoxia |
| J96.92 | (above), but with hypercapnia |
| J06.9 | Acute Respiratory Infection, Unspecified |
| R09.2 | Respiratory arrest |

**eAppendix Table 8**. Outpatient Clinic Visits for Reports of Cough

|  | **Dec** | | | | **Jan** | | | | **Feb** | | | |
| --- | --- | --- | --- | --- | --- | --- | --- | --- | --- | --- | --- | --- |
|  | Visit | case | % | case per 1,000 | Visit | case | % | case per 1,000 | Visit | case | % | case per 1,000 |
| 2014 - 2015 | 102,764 | 273 | 0.27% | 3 | 112,503 | 375 | 0.33% | 3 | 99,565 | 281 | 0.28% | 3 |
| 2015 - 2016 | 125,772 | 419 | 0.33% | 3 | 128,529 | 468 | 0.36% | 4 | 136,788 | 612 | 0.45% | 4 |
| 2016 - 2017 | 127,993 | 437 | 0.34% | 3 | 141,626 | 616 | 0.43% | 4 | 136,001 | 618 | 0.45% | 5 |
| 2017 - 2018 | 126,448 | 460 | 0.36% | 4 | 157,345 | 728 | 0.46% | 5 | 141,893 | 482 | 0.34% | 3 |
| 2018 - 2019 | 133,395 | 413 | 0.31% | 3 | 164,487 | 617 | 0.38% | 4 | 148,791 | 605 | 0.41% | 4 |
| 2019 - 2020 | 156,400 | 774 | 0.49% | 5 | 185,428 | 1142 | 0.62% | 6 | 169,859 | 1022 | 0.60% | 6 |
| **Estimated excess cases (per 1,000 visits)** | 1.3 - 1.7 | | | | 1.5 - 2.2 | | | | 1.5 - 2.2 | | | |

December:

Compared with the highest historical value: .49% - .36% = .13%; .13% x 156,400 = 205

Compared with the average historical value: .49% - .33% = .16%; .17% x 156,400 = 269

January

Compared with the highest historical value: .62% - .46% = .16%; .16% x 185,428 = 284

Compared with the average historical value: .62% - .39% = .23%; .23% x 185,428 = 411

February

Compared with the highest historical value: .60% - .45% = .15%; .15% x 169,859 = 250

Compared with the average historical value: .60% - .39% = .21%; .21% x 169,859 = 366

**eAppendix Table 9**. Emergency Room Visits for Reports of Cough

|  | **Dec** | | |  | **Jan** | | | | **Feb** | | | |
| --- | --- | --- | --- | --- | --- | --- | --- | --- | --- | --- | --- | --- |
|  | Visit | case | % | case per 1,000 | Visit | case | % | case per 1,000 | Visit | case | % | case per 1,000 |
| 2014 - 2015 | 7730 | 198 | 2.56% | 26 | 8815 | 393 | 4.46% | 45 | 7582 | 262 | 3.46% | 35 |
| 2015 - 2016 | 8224 | 271 | 3.30% | 33 | 8927 | 355 | 3.98% | 40 | 8826 | 508 | 5.76% | 58 |
| 2016 - 2017 | 8516 | 296 | 3.48% | 35 | 8844 | 412 | 4.66% | 47 | 8145 | 364 | 4.47% | 45 |
| 2017 - 2018 | 9105 | 452 | 4.96% | 50 | 9560 | 558 | 5.84% | 58 | 8357 | 419 | 5.01% | 50 |
| 2018 - 2019 | 8464 | 369 | 4.36% | 44 | 8898 | 448 | 5.03% | 50 | 8193 | 446 | 5.44% | 54 |
| 2019 - 2020 | 8608 | 484 | 5.62% | 56 | 9505 | 708 | 7.45% | 74 | 8635 | 516 | 5.98% | 60 |
| **Estimated excess cases (per 1,000 visits)** | 6.6 - 18.9 | | | | 16.1 - 26.6 | | | | 2.2 - 11.5 | | | |

December:

Compared with the highest historical value: 5.62%– 4.96% = 0.66%; .66% x 8608= 57

Compared with the average historical value: 5.62%– 3.73% = 1.89%; 1.89% x 8608= 163

January

Compared with the highest historical value: 7.45% - 5.84% = 1.61%; 1.61% x 9505= 153

Compared with the average historical value: 7.45% - 4.79% = 2.66%; 2.66% x 9505= 252

February

Compared with the highest historical value: 5.98% - 5.76% = 0.22%; 0.2% x 8635 = 19

Compared with the average historical value: 5.98% - 4.83% = 1.15%; 1.15% x 8635 = 99

**eAppendix Table 10.** Hospitalization Data

**Hospitalizations for A. Respiratory tract diagnoses (Using ICD codes from Chow, et al, 2020)**

|  | **Dec** | | | | **Jan** | | | | **Feb** | | | |
| --- | --- | --- | --- | --- | --- | --- | --- | --- | --- | --- | --- | --- |
|  | Stay | case | % | case per 1,000 | Stay | case | % | case per 1,000 | Stay | case | % | case per 1,000 |
| 2014 - 2015 | 3647 | 491 | 13.46% | 135 | 3838 | 614 | 16.00% | 160 | 3531 | 498 | 14.10% | 141 |
| 2015 - 2016 | 3559 | 497 | 13.96% | 140 | 3731 | 530 | 14.21% | 142 | 3635 | 567 | 15.60% | 156 |
| 2016 - 2017 | 3671 | 523 | 14.25% | 142 | 3780 | 614 | 16.24% | 162 | 3380 | 549 | 16.24% | 162 |
| 2017 - 2018 | 3523 | 587 | 16.66% | 167 | 3791 | 637 | 16.80% | 168 | 3326 | 521 | 15.66% | 157 |
| 2018 - 2019 | 3366 | 514 | 15.27% | 153 | 3818 | 584 | 15.30% | 153 | 3462 | 514 | 14.85% | 148 |
| 2019 - 2020 | 3325 | 581 | 17.47% | 175 | 3548 | 700 | 19.73% | 197 | 3030 | 568 | 18.75% | 187 |
| **Estimated excess cases (per 1,000 visits)** | 8.1 - 27.5 | | | | 29.3 - 40.2 | | | | 25.0 - 34.5 | | | |

**Estimated Excess in Patients Admitted with Respiratory Tract Diagnoses**

December: 27, 92

January: 104, 143

February: 76, 105

**Hospitalizations for B. Any Pneumonia**

|  | **Dec** | | | | **Jan** | | | | **Feb** | | | |
| --- | --- | --- | --- | --- | --- | --- | --- | --- | --- | --- | --- | --- |
|  | Stay | case | % | case per 1,000 | Stay | case | % | case per 1,000 | Stay | case | % | case per 1,000 |
| 2014 - 2015 | 3647 | 303 | 8.31% | 83 | 3838 | 429 | 11.18% | 112 | 3531 | 322 | 9.12% | 91 |
| 2015 - 2016 | 3559 | 308 | 8.65% | 87 | 3731 | 330 | 8.84% | 88 | 3635 | 356 | 9.79% | 98 |
| 2016 - 2017 | 3671 | 328 | 8.93% | 89 | 3780 | 383 | 10.13% | 101 | 3380 | 352 | 10.41% | 104 |
| 2017 - 2018 | 3523 | 400 | 11.35% | 114 | 3791 | 447 | 11.79% | 118 | 3326 | 325 | 9.77% | 98 |
| 2018 - 2019 | 3366 | 278 | 8.26% | 83 | 3818 | 359 | 9.40% | 94 | 3462 | 308 | 8.90% | 89 |
| 2019 - 2020 | 3325 | 347 | 10.44% | 104 | 3548 | 439 | 12.37% | 124 | 3030 | 329 | 10.86% | 109 |
| **Estimated excess cases (per 1,000 visits)** | (-9.2) - 13.3 | | | | 5.8 - 21.0 | | | | 4.4 - 12.6 | | | |

**Estimated Excess in Patients Admitted for Pneumonia:**

December: -31, 44

January: 21, 75

February: 13, 38

**Hospitalizations for C. Acute Respiratory Failure**

|  | **Dec** | | | | **Jan** | | | | **Feb** | | | |
| --- | --- | --- | --- | --- | --- | --- | --- | --- | --- | --- | --- | --- |
|  | Stay | case | % | case per 1,000 | Stay | case | % | case per 1,000 | Stay | case | % | case per 1,000 |
| 2014 - 2015 | 3647 | 212 | 5.81% | 58 | 3838 | 244 | 6.36% | 64 | 3531 | 224 | 6.34% | 63 |
| 2015 - 2016 | 3559 | 248 | 6.97% | 70 | 3731 | 253 | 6.78% | 68 | 3635 | 259 | 7.13% | 71 |
| 2016 - 2017 | 3671 | 273 | 7.44% | 74 | 3780 | 299 | 7.91% | 79 | 3380 | 255 | 7.54% | 75 |
| 2017 - 2018 | 3523 | 274 | 7.78% | 78 | 3791 | 294 | 7.76% | 78 | 3326 | 262 | 7.88% | 79 |
| 2018 - 2019 | 3366 | 334 | 9.92% | 99 | 3818 | 338 | 8.85% | 89 | 3462 | 324 | 9.36% | 94 |
| 2019 - 2020 | 3325 | 363 | 10.92% | 109 | 3548 | 416 | 11.72% | 117 | 3030 | 359 | 11.85% | 118 |
| **Estimated excess cases (per 1,000 visits)** | 9.9 - 33.3 | | | | 28.7 - 41.9 | | | | 24.9 - 42.0 | | | |

**Estimated Excess in Patients Admitted with Acute Respiratory Failure**

December: 33, 111

January: 102, 149

February: 127, 75

**eAppendix Table 11.** Hospitalizations for any respiratory tract disease or pneumonia by years of winter season.

| **Calendar**  **Year** |  | **Hospitalizations** | | | | | | | | |
| --- | --- | --- | --- | --- | --- | --- | --- | --- | --- | --- |
|  | **Total Stay** | **Any Respiratory Tract** | | |  | **Pneumonia** | | | | |
|  |  | **N** | **%** | **cases per 1,000** |  | **N** | **%** | | **cases per 1,000** | |
| **2014 - 2015** | 11,016 | 1,603 | 14.55% | 145.5 |  | 1,054 | 9.57% | | 96 | |
| **2015 - 2016** | 10,925 | 1,594 | 14.59% | 145.9 |  | 994 | 9.10% | | 91 | |
| **2016 - 2017** | 10,831 | 1,686 | 15.57% | 155.7 |  | 1,063 | 9.81% | | 98 | |
| **2017 - 2018** | 10,640 | 1,745 | 16.40% | 164.0 |  | 1,172 | 11.02% | | 110 | |
| **2018 - 2019** | 10,646 | 1,612 | 15.14% | 151.4 |  | 945 | 8.88% | | 89 | |
| **2019 - 2020** | 9,903 | 1,849 | 18.67% | 186.7 |  | 1,115 | 11.26% | | 113 | |
| **Estimated excess cases per 1,000 visits*** | **22.7, 34.2** | | | |  | **2.4, 15.8** | |  | |  |
| **Estimated excess cases*** |  | **207, 339** | |  |  | **4, 157** | | |  | |

*Two methods were used to estimate the excess cases per 1,000 visits and excess cases in the 2019-2020 winter season (December 2019, January 2020, and February 2020) compared to previous seasons. First, for a conservative estimate for excess cases per 1,000 visits, using percentages we subtracted the maximum of the five previous seasons with the current season. Second, we subtracted the average of the five previous seasons from the current season. For both methods, we multiplied the excess percentage by the total number of patient visits in the current season to estimate the excess cases.

**eFigure 4.** Scatter plot of daily data with fitted LOESS smoothing lines for each year

1. **outpatient clinic visits for cough.**

**
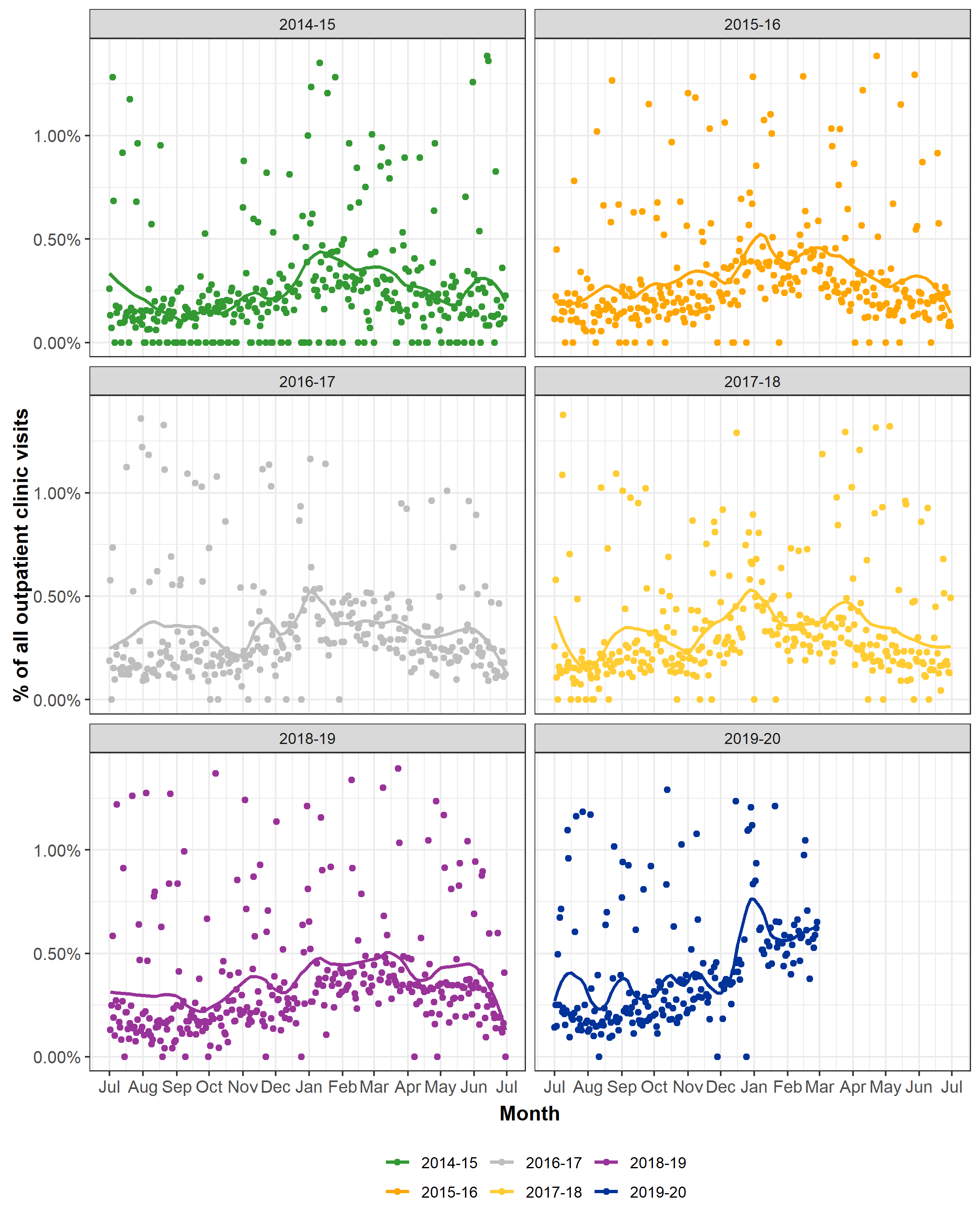
**

1. **Emergency department visits for cough**

**
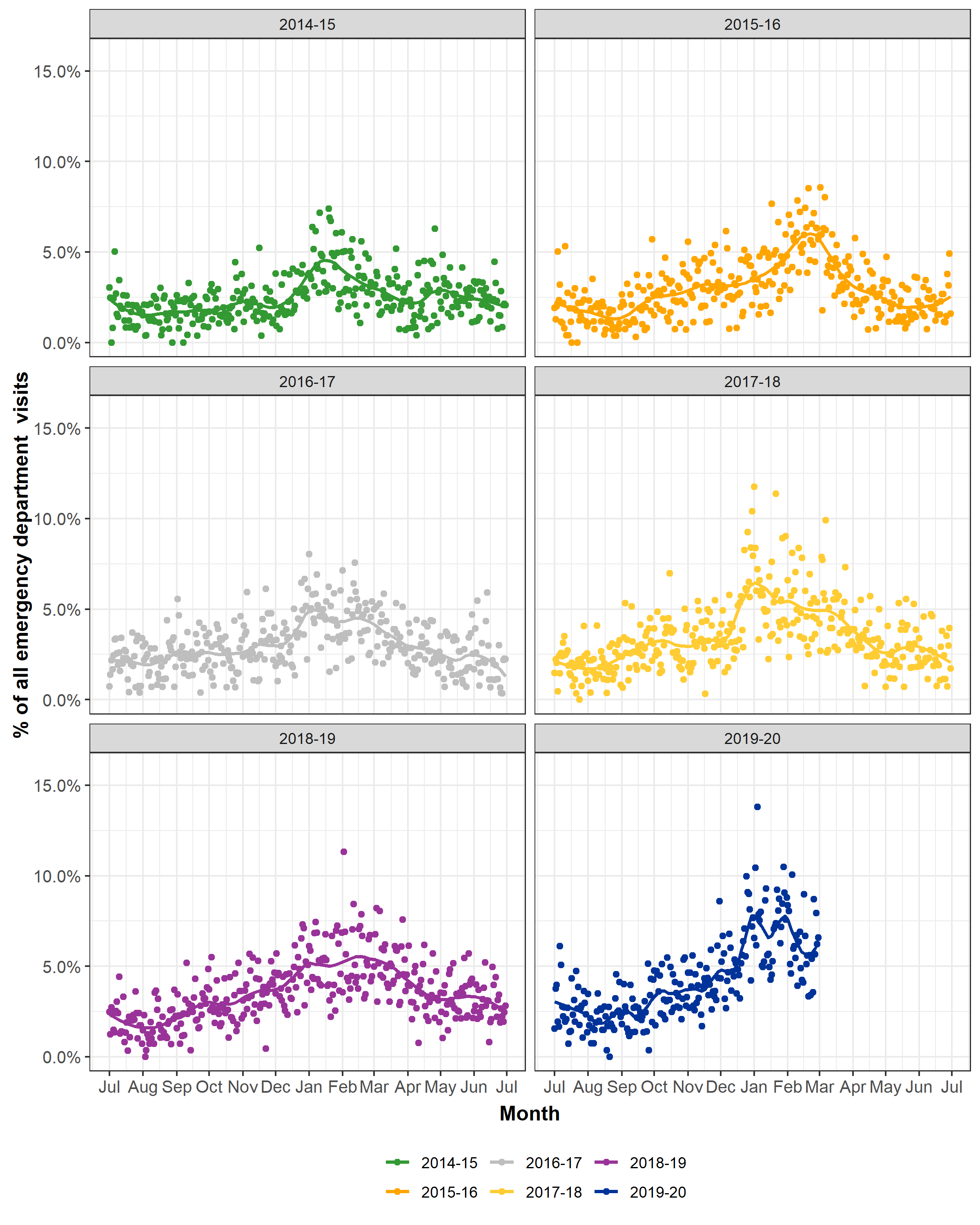
**

1. **Hospitalization Data**

**Set A. Respiratory tract diagnoses (Using ICD codes from Chow, et al 2020)**

**
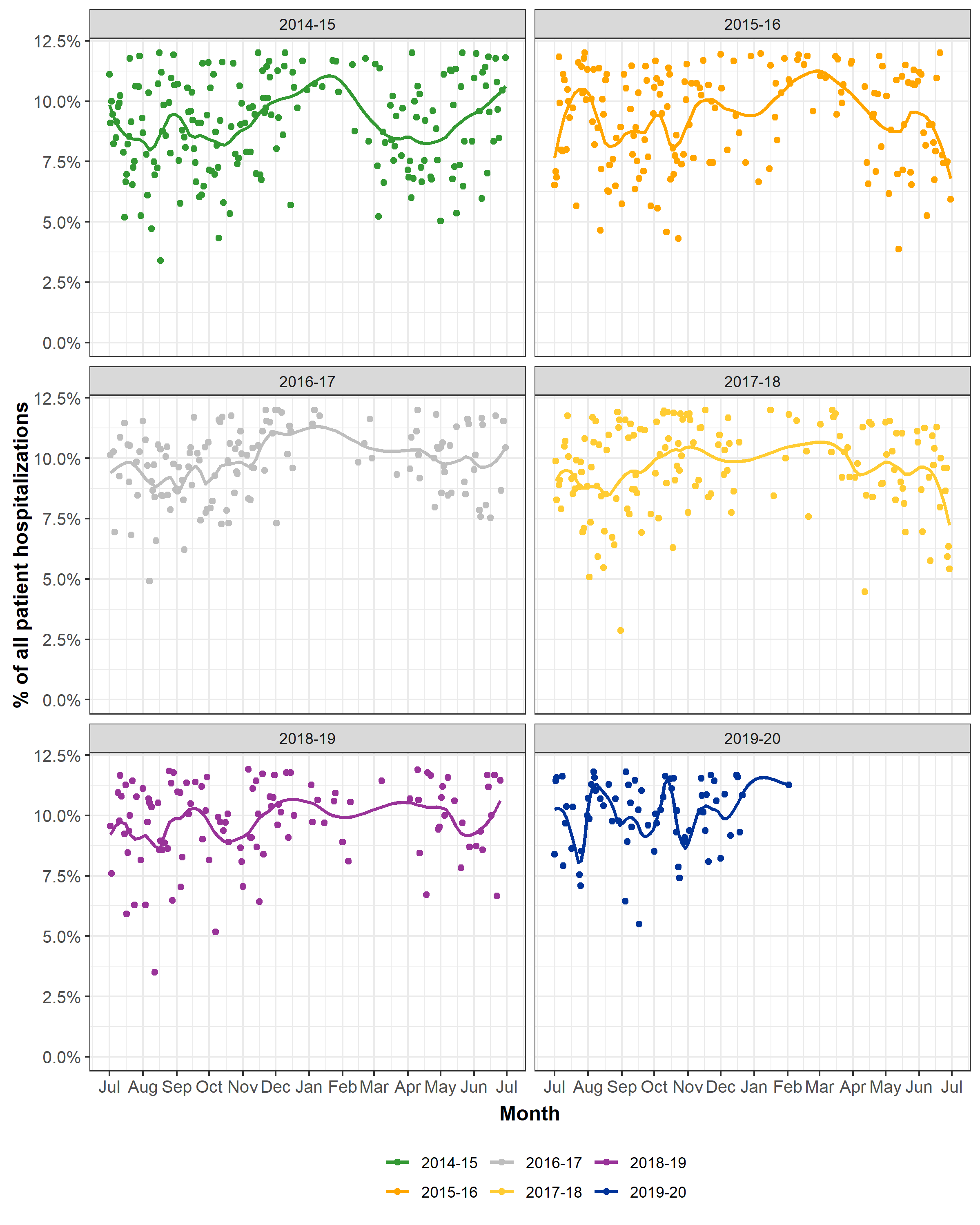
**

**Hospitalization Data**

**Set B. Pneumonia**

**
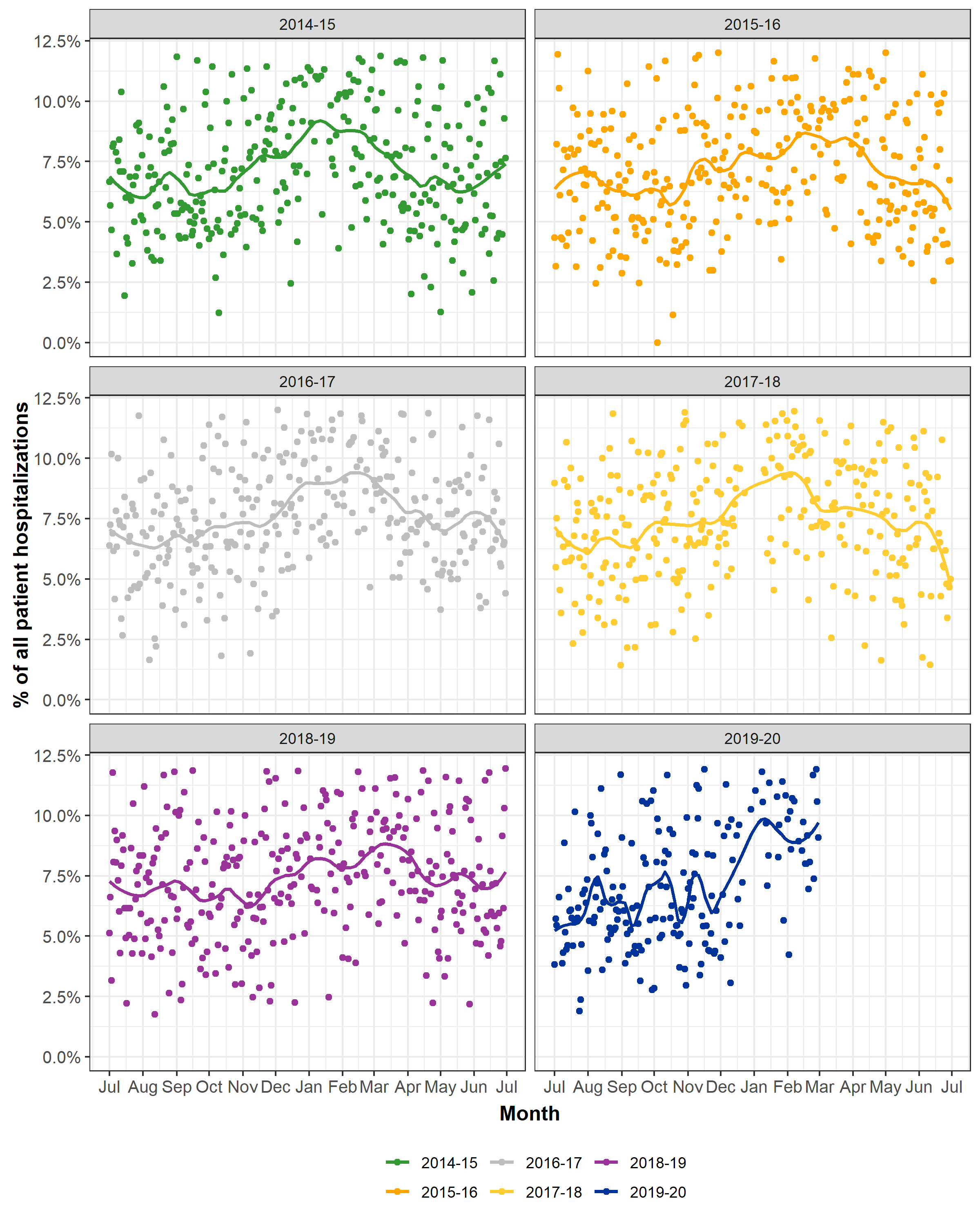
**

**Hospitalization Data**

**Set C. Acute Respiratory Failure**

**
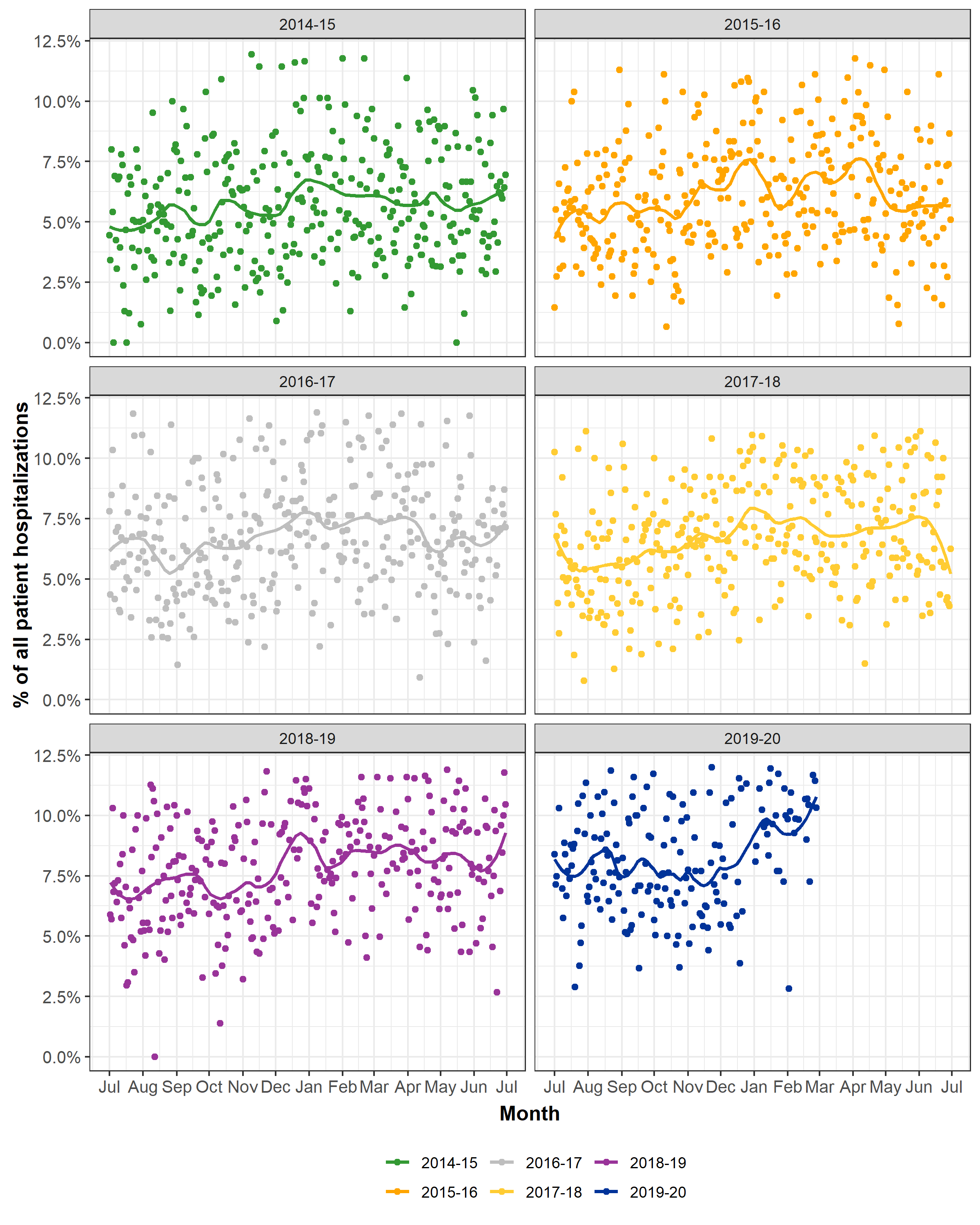
**

**eFigure 5.** Outpatient Visit Data by Insurance Status


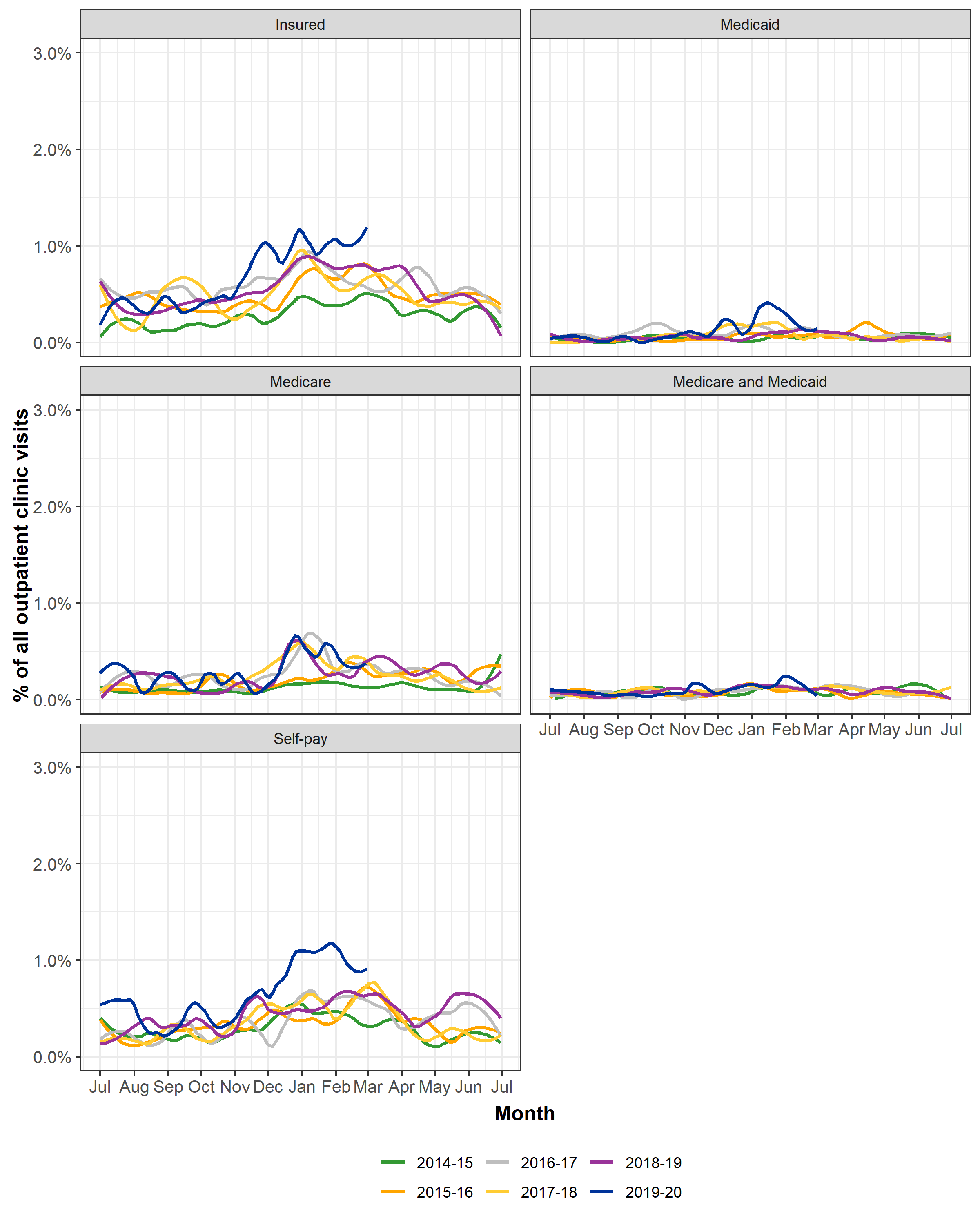


**eFigure 6.** Sensitivity test – Outpatient visits for cough by selected clinics (consistently operated from 2014 to 2020)

**
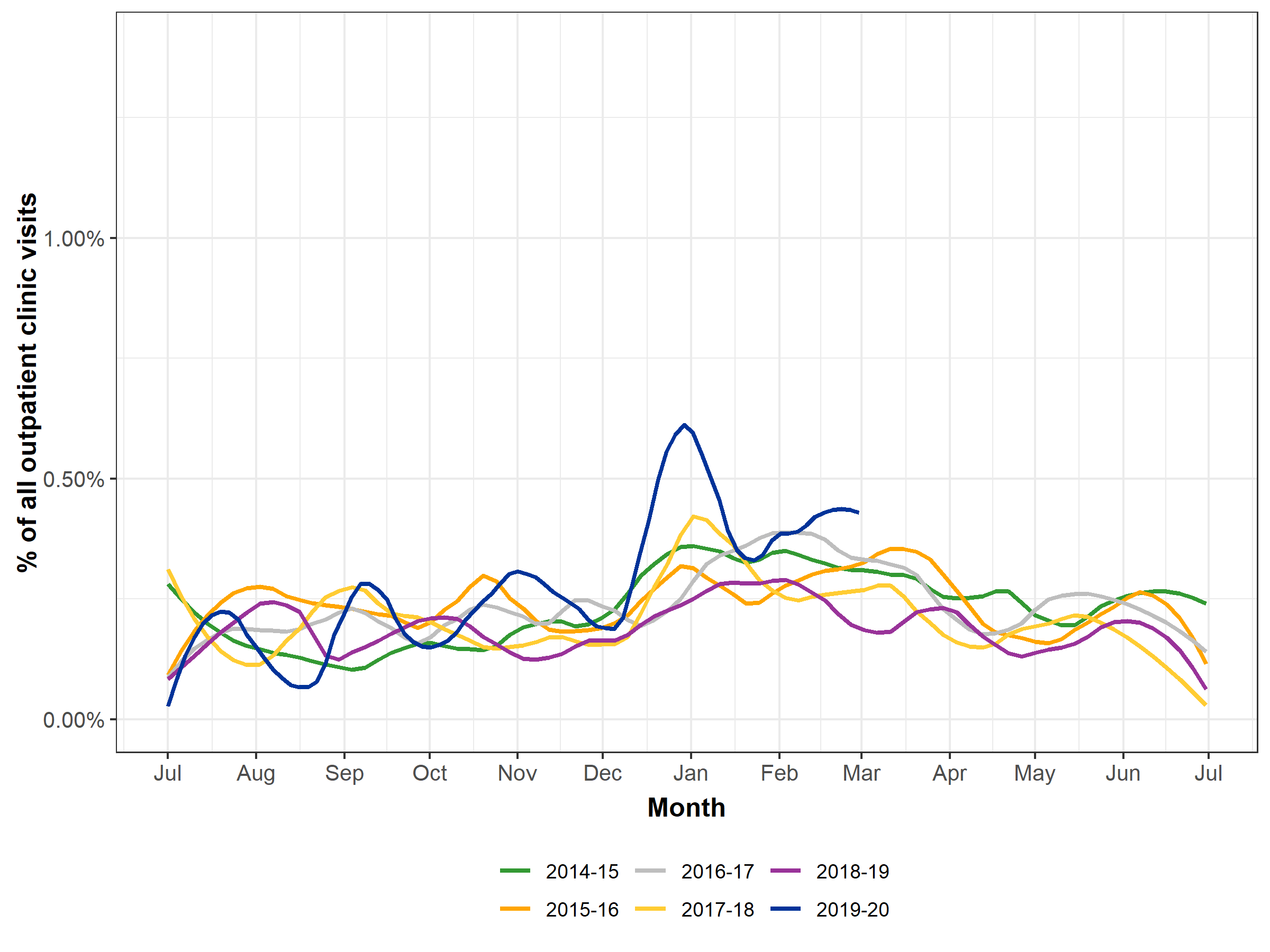
**

|  | **Dec** | | | | **Jan** | | | | **Feb** | | | |
| --- | --- | --- | --- | --- | --- | --- | --- | --- | --- | --- | --- | --- |
|  | Visit | Cough# | Cough(%) | case per 1,000 | Visit | Cough# | Cough(%) | case per 1,000 | Visit | Cough# | Cough(%) | case per 1,000 |
| 2014 - 2015 | 84,523 | 211 | 0.25% | 2 | 92,394 | 282 | 0.31% | 3 | 81,845 | 231 | 0.28% | 3 |
| 2015 - 2016 | 87,607 | 213 | 0.24% | 2 | 89,771 | 239 | 0.27% | 3 | 95,082 | 267 | 0.28% | 3 |
| 2016 - 2017 | 86,662 | 212 | 0.24% | 2 | 94,907 | 302 | 0.32% | 3 | 91,181 | 313 | 0.34% | 3 |
| 2017 - 2018 | 79,427 | 207 | 0.26% | 3 | 98,172 | 356 | 0.36% | 4 | 88,662 | 241 | 0.27% | 3 |
| 2018 - 2019 | 81,440 | 148 | 0.18% | 2 | 99,757 | 182 | 0.18% | 2 | 89,573 | 173 | 0.19% | 2 |
| 2019 - 2020 | 84,534 | 308 | 0.36% | 4 | 100,141 | 461 | 0.46% | 5 | 90,487 | 420 | 0.46% | 5 |
| **Estimated excess cases (per 1,000 visits)** | 1.3 - 1.7 | | | | 1.5 - 2.2 | | | | 1.5 - 2.2 | | | |

**eFigure 7.** Sensitivity test – Emergency Department visits for cough by Insurance Coverage

**
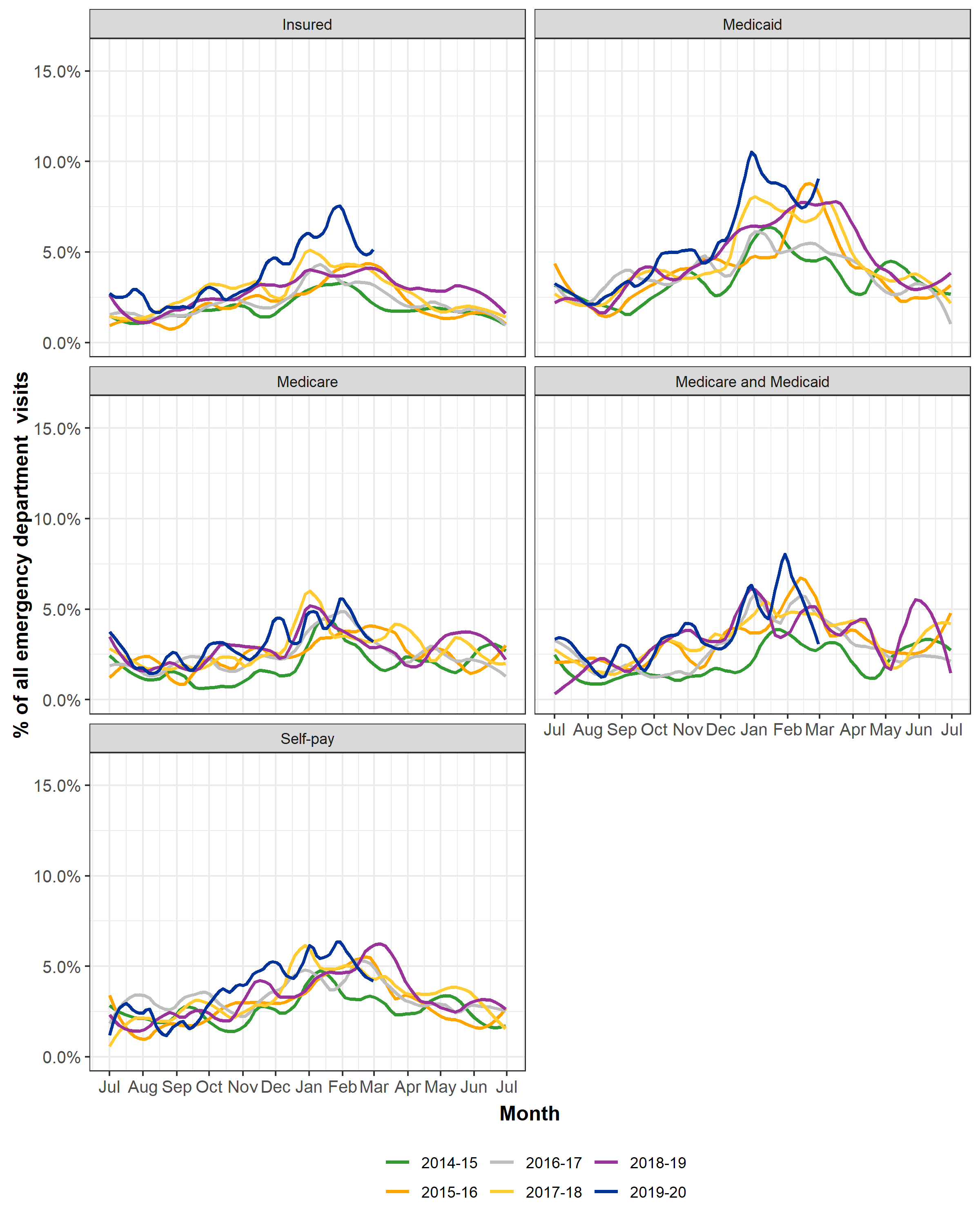
**

**eFigure 8.** Popularity of Web Searches for “Cough” in the U.S. (blue line) and News Searches for “Coronavirus/COVID” in the U.S. (orange line) for November 2019 to March 31, 2020, with historical average of Web Searches for “Cough” in the U.S. across the previous four years (black line). *


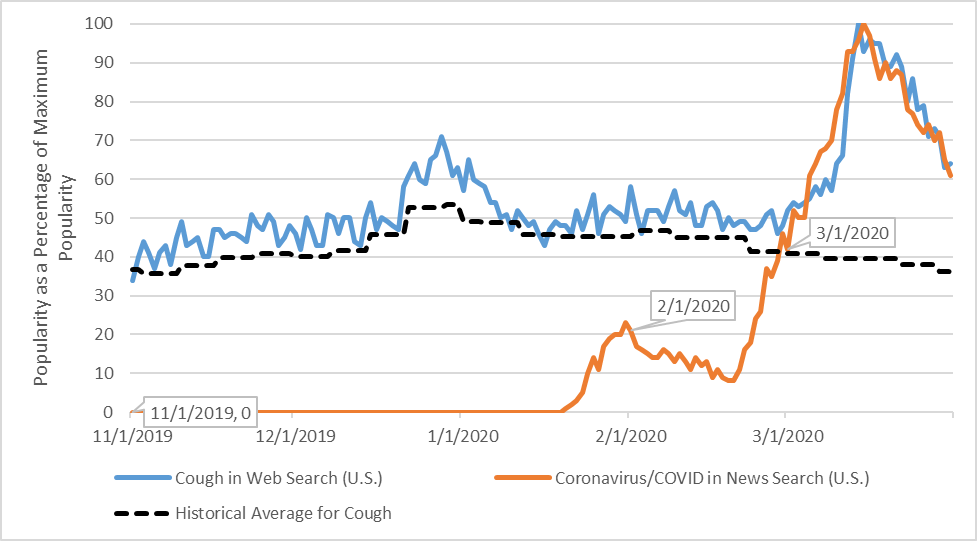


*Note: Daily values are presented for web searches for “Cough” in the current year (blue line). Weekly values are presented for historical averages of web searches for “Cough”. Historical averages were calculated using the same week of the year across the prior four years (November 2015 to March 2019). Searches for “Coronavirus/COVID” represent U.S. searches for “coronavirus”, “covid”, or “covid19” in the context of news.
